# Factors associated with COVID-19 vaccine intentions during the COVID-19 pandemic; a systematic review and meta-analysis of cross-sectional studies

**DOI:** 10.1186/s12889-022-14029-4

**Published:** 2022-09-02

**Authors:** Emily Terry, Sapphire Cartledge, Sarah Damery, Sheila Greenfield

**Affiliations:** 1 University of Birmingham, College of Medical and Dental Sciences, University of Birmingham, Edgbaston, Birmingham, B15 2TT England; 2 Institute of Applied Health Research, College of Medical and Dental Sciences, University of Birmingham, Edgbaston, Birmingham, B15 2TT England

**Keywords:** Vaccine, Vaccine intentions, Vaccine hesitancy, COVID-19, Coronavirus, Pandemic, Health behaviour, Public Health, Attitudes to Health

## Abstract

**Background:**

A high COVID-19 vaccine uptake is essential to achieve herd immunity to combat the current strain of COVID-19 and potential future variants. This review aimed to identify factors associated with public intention to receive COVID-19 vaccines until February 2021 to provide accessible data to policymakers to inform framing and targeting of messages designed to optimise vaccine uptake.

**Methods:**

Medline, Embase, CINAHL, PsycINFO, PsycARTICLES, Sociological Abstracts and Applied Social Sciences Index and Abstracts were searched for cross-sectional studies reporting data regarding COVID-19 vaccine intentions, published between 01/01/2020 and 12/02/2021. Title/abstract and full-text screening were performed independently by two authors. The Appraisal Tool for Cross-sectional Studies (AXIS) was used to assess bias and quality. Both random-effects meta-analysis and narrative synthesis were used to describe vaccine intentions and associated factors. A subgroup analysis assessing the impact of sex, sampling method and time of survey on COVID-19 vaccine intention was performed.

**Results:**

Searches identified 4739 studies, and 23 cross-sectional studies were deemed eligible for the review; 22 used online surveys and one used a mixed-methods study design. Eighteen surveys were conducted in the first half of 2020 and five were conducted in the latter half of 2020. Fifteen countries were represented, with the most common being the United States (*n* = 4) and the United Kingdom (*n* = 4) sampling 41,403 participants across all surveys. Most studies employed convenience sampling and 11 non-responder rates raised concerns over non-response bias. From the 18 studies included in the meta-analysis, the pooled proportion of survey participants willing to receive the COVID-19 vaccine was 73.3% (*n* = 18, 95% Confidence Interval 64.2 to 81.5%, I^2^ = 99.7%). Factors associated with a higher COVID-19 vaccine acceptance included greater perceived risk of COVID-19, lower level of perceived vaccine harm, higher educational attainment and household income, older age, being of White ethnicity and male sex.

**Conclusions:**

There was a high willingness to receive the COVID-19 vaccine which was influenced by sociodemographic factors and risk perceptions. The findings suggest future research should explore reasoning behind vaccine intentions for different sociodemographic groups to allow targeted communication strategies to be formulated by public health agencies.

**Registration:**

PROSPERO Registration Number: CRD42021239134.

**Supplementary Information:**

The online version contains supplementary material available at 10.1186/s12889-022-14029-4.

## Background

Since coronavirus disease 2019 (COVID-19) was first identified in Wuhan, China in December 2019 [1], there have been numerous coronavirus case surges around the globe [2]. The development of effective COVID-19 vaccines has given hope to the global community, with the vaccine rollout marking a ‘turning point’ in the battle against coronavirus [3].

Mass vaccination programmes aim to vaccinate a large proportion of the population so that disease transmission is slowed and vulnerable individuals who cannot be vaccinated are still protected [4]. This phenomenon is known as herd immunity and can only be achieved when a substantial proportion of the population is vaccinated [5]. The threshold to achieve herd immunity against COVID-19 is estimated at between 60 and 70% [4]. However, due to the viral nature of COVID-19, mutations are inevitable and the main ‘Delta’ variant in India has taken over as the dominant strain in many countries [6]. New variants may be more transmissible which will require a higher herd immunity threshold, and/or more likely to cause severe infection [7]. The situation is constantly evolving hence a high vaccine uptake is essential to combat the current dominant strain of COVID-19 and any potential future strains [8].

Global public trust in governments has rapidly declined throughout the pandemic, with the Edelman Trust Barometer reporting a significant decline in trust between both the Chinese and American government and their own citizens between May 2020 and January 2021 [9]. Together with the increasingly prominent role of social media, pandemics are a breeding ground for fearmongering and rumours to circulate [10]. Online misinformation circulating on social media regarding COVID-19 is a growing problem [11]. In the case of COVID-19 (as in past pandemics), the dissemination of vaccine misinformation has been particularly prevalent in fuelling a growing anti-vaccination movement [12, 13]. A recent analysis of social media identified that 39% of online rumours regarding the COVID-19 pandemic were about the COVID-19 vaccine, with 76% of such rumours reported to be false [14].

The term ‘vaccine hesitancy’ refers to a delayed acceptance or complete refusal of a vaccine [15]. The effects of vaccine hesitancy can be devasting. The diphtheria-tetanus-pertussis (DTP) vaccine was routinely used in the UK for over 20 years [16]. However, following the publication of case-series linking the vaccine to a rare neurological side-effect, there was a dramatic fall in immunisation rates against DTP from 77 to 33% [16, 17]. This was followed by three whooping-cough epidemics in the UK [17]. Therefore, historic evidence suggests that uncertain times can increase individual and societal vaccine hesitancy.

Many factors may contribute to vaccine hesitancy [15]. A systematic review of adults aged 65 years and older in the United States of America (USA) identified female sex, older age, higher education, higher household income and White ethnicity all increase the likelihood of seasonal influenza vaccination uptake [18]. It is currently unclear whether COVID-19 vaccine intentions are influenced by the same trend in socio-demographic factors. Public Health England (PHE) reported large disparities in mortality and morbidity risks of COVID-19 infection between different sociodemographic groups, with more deprived areas and ethnic minority individuals (particularly Black ethnic groups) shown to be at a higher risk [19]. A rapid national assessment in 2021 in the United States highlighted that less educated, minority groups had a higher rate of COVID-19 vaccine hesitancy [20]. Arguably, these groups would benefit the most from the COVID-19 vaccine. A theory-based analysis of COVID-19 vaccine hesitancy among African Americans in the United States in July-August 2021 discovered that 48.6% of African-Americans had not been COVID-19 vaccinated and expressed vaccine hesitancy and the rates of vaccine hesitancy were significantly higher amongst younger individuals [21]. This highlights both similarities and differences between traditional socio-demographic trends in vaccine hesitancy and COVID-19 vaccine hesitancy.

Since the beginning of the COVID-19 pandemic, several systematic reviews have been conducted into COVID-19 vaccine uptake and adherence [22–26].

Lin et al. (December 2020) evaluated 126 cross-sectional surveys of vaccine intentions dating from February to October 2020 [22]. A narrative synthesis of results described a declining trend in vaccine intention and highlighted socioeconomic and ethnic issues pertaining to vaccine availability. Due to the dynamic nature of public opinion during the pandemic, the review recommends continuous monitoring of vaccine intentions, especially following the introduction of mass-vaccination programmes. A recent study has suggested that traditional sociodemographic factors may not be effective predictors of COVID-19 vaccine uptake [27]. Therefore, it is not unreasonable to suggest that traditional public health campaigns may be ineffective at boosting COVID-19 vaccine intentions. The continuous monitoring of COVID-19 vaccine intentions among different socioeconomic groups is essential in order to design effective public health campaigns to tackle hesitancy towards the COVID-19 vaccine specifically.

Robinson et al. (December 2020) published a smaller review of 28 international cross-sectional studies and survey dates ranged from March-October [23]. The review reported a high rate of vaccine intention across survey participants (72.9% of total participants were prepared to have a COVID-19 vaccine, 95% confidence interval (CI) 66.6 to 78.4%, I^2^ = 99.6%) and found a declining trend in vaccine willingness over time. It searched only two online databases and only one author conducted all stages of screening and data extraction. Limited database searching can introduce selection bias [28] and the absence of conventional double screening can result in the omission of key studies [29]. This review included surveys with a large sample size only (*n* ≥ 100) [23], compared to Lin et al.’s review which included surveys with smaller sample sizes [22]. Overall, the review conducted by Robinson et al. provides a systematic summary of the global populations’ acceptance towards the COVID-19 vaccine up until October 2020 [23]. Since the rollout of COVID-19 vaccine programmes, booster doses of the vaccine have emerged to provide continuous long-term protection from the virus. A cross-sectional study among the American population suggested a strong predictor of booster hesitancy was primary COVID-19 vaccine status; the vaccine-booster-hesitant groups were almost 5 times more likely to be unvaccinated [30]. This highlights the importance of tackling hesitancy towards the primary vaccine dose, due to the apparent domino effect this may have on subsequent booster doses. Given the emergence of breakthrough infections and new variants, it is currently unclear how long the population will be required to receive booster doses to maintain immunity and control the virus. Therefore, it is essential that vaccine hesitancy towards the COVID-19 vaccine is understood, before we can begin to improve booster vaccine acceptance.

Given the rapidly evolving course of the pandemic, ongoing research is needed to reflect the changing evidence base and summarise public opinion later in the pandemic. Therefore, this systematic review will provide an updated summary of public opinion towards the COVID-19 vaccine around the globe up until February 2021, with the intention of providing readily accessible data to policymakers.

## Aims

This review aimed to assess: (1) general population intention to receive the COVID-19 vaccine around the world up until February 2021 and changes over time; (2) factors associated with COVID-19 vaccine acceptance; (3) reasons behind individuals’ vaccination intention.

## Methods

### Eligibility criteria

As recommended by the Cochrane Collaboration, the research question and eligibility criteria were framed using the SPIDER search tool, to maintain a focused review (Additional file 1) [31].

### Search methods

This review was developed and structured in line with the 2020 Preferred Reporting Items for Systematic Reviews (PRISMA) guidelines and the protocol was registered in PROSPERO (Registration Number: CRD42021239134) [32].

#### Information sources

A literature search of Medline (Ovid) [33], Embase (Ovid) [34] and CINAHL (EBSCO) [35] was undertaken by one author (ET). Specialist social sciences databases were also searched: APA PsycINFO (Ovid) [36], APA PsycARTICLES (Ovid) [37], Sociological Abstracts (ProQuest) [38] and Applied Social Sciences Index and Abstracts (ASSIA, ProQuest) [39].

#### Search strategy

Search strategy development was guided by a librarian specialist. Several scoping searches were conducted on Medline [33] and Embase [34] to identify relevant literature and understand any differences in standardised subject terms across the databases. Development of search terms were guided by the results of the scoping review. The search strategy was subsequently piloted using Medline [33] and refined until all key papers identified in the scoping review were retrieved from the first 100 search results. A combination of text words and standardised subject terms were used, adjusted for each database, to avoid missing key literature.

For the purpose of this review, studies investigating vaccine intentions were included, with vaccine hesitancy defined as “a delay in acceptance or refusal of vaccination despite availability of vaccination services” [40] and vaccine acceptance defined as “outcome behaviour resulting from a complex decision-making process that can be potentially influenced by a wide range of factors” [40]. We searched explicitly for papers that included data on these terms, using the search terms ‘COVID-19’, ‘Pandemics’, ‘Intention’, ‘Attitude to Health’, ‘Mass Vaccination’, ‘Vaccination Refusal’, ‘Anti-Vaccination Movement’ and ‘Vaccination’. Search terms were combined with the Boolean operators ‘AND’ or ‘OR’, the explosion function was used where possible, and truncation was utilised to capture all alternative spellings of the terms. Searches were conducted on 12/02/2021. Full detailed searches, tailored to each database can be found in Additional file 2.

#### Limits

Date of publication was limited from 1st January 2020 to the day the search was undertaken. The study-type was limited to cross-sectional studies. No geographical limits were applied, but only studies published in English were eligible.

#### Data management

Endnote was used to store references and remove duplicates automatically [41]. The web-based reviewing platform Rayann was used for title/abstract and full-text screening [42].

#### Selection process

Title and abstract screening were performed independently by two authors (ET and SC), who both performed full-text assessment of potentially eligible studies (Additional file 1). All discrepancies in inclusion/exclusion decisions at both stages of screening were discussed by ET and SC over the online video platform Zoom initially and with SD as a third reviewer when a decision could not be made [43]. As screening was undertaken by two novice reviewers, inter-rater reliability was measured using Cohen’s Kappa coefficient at both stages, with a Kappa value of > 0.6 deemed to represent substantial agreement [44].

### Data extraction and synthesis

#### Data collection process

A data extraction form (Additional file 6) was developed and piloted prior to use. Data were extracted by ET and a random sample of 10% of studies was co-assessed by SD, to minimise data extraction errors. Any differences of opinions were discussed, to ensure that all relevant data were extracted. For each study, study characteristics were extracted including study design, sample size, location, survey timescale, method of recruitment, participant demographics, validation and standardisation of the survey instruments, the specific survey questions used to capture attitudes towards the COVID-19 vaccine, response scales, recorded proportions of vaccine intentions and any other relevant information.

#### Risk of Bias and quality assessment

Study quality and risk of bias were assessed using the Appraisal Tool for Cross-Sectional Studies (AXIS) (Additional file 7) [45] which was piloted for suitability on two studies. ET assessed all studies, with 10% co-assessed independently by a second author (SC). Again, Cohen’s Kappa coefficient was calculated to test inter-rater reliability.

#### Data synthesis

Data were summarised using narrative syntheses and meta-analyses as appropriate. Where response proportions were represented as raw numbers, data were converted to percentages of total survey participants in each included study.

All studies were assessed to determine whether it was appropriate to statistically combine the survey findings. Guided by Lin at al., surveys were excluded from analysis if questions included persuasive or influencing language; if they included phrases similar to ‘if a safe and effective vaccine was available’ [22]. The remaining surveys were included in a random-effects meta-analysis using the ‘metaprop’ command [46], to estimate the proportion of total survey participants reporting vaccine acceptance (including 95% confidence intervals). For studies that included a 5-point Likert scale, vaccine acceptance represented the proportions of both ‘strongly agree’ and ‘agree’ responses. Percentage proportions were presented using forest plots, including statistical heterogeneity. Substantial heterogeneity (I^2^ > 85%) was expected due to the nature of the survey outcome; an individual’s decision on vaccine uptake may be influenced by multiple and potentially overlapping factors simultaneously. Publication bias was not assessed; instead, sub-group analyses of the meta-regression by sample size (*n* < 1000 vs *n* ≥ 1000) and sampling method (non-probability vs probability sampling) were performed.

Across studies that included four response categories (variations of ‘strongly agree’, ‘agree’, ‘disagree’ and ‘strongly disagree’), the mean proportion of responses in each category were compared. Across studies that included a hesitant response category (variations of ‘maybe’), the overall proportion of survey participants reporting COVID-19 vaccine hesitant and improbable/very improbable were compared.

For all studies, the influence of health beliefs and sociodemographic variables (age, gender, ethnicity, education, and income level) on vaccine acceptance, and reasons for vaccine hesitancy were summarised narratively. Of the studies included in the meta-regression, further sub-group analyses of participants reporting vaccine acceptance by gender and time of survey were performed on studies that reported the relevant data.

Statistical significance for all analyses was set at the 5% level (*p* = 0.05) and all statistical analyses were conducted using STATA16 [46].

## Results

### Study selection

**Fig. 1:**
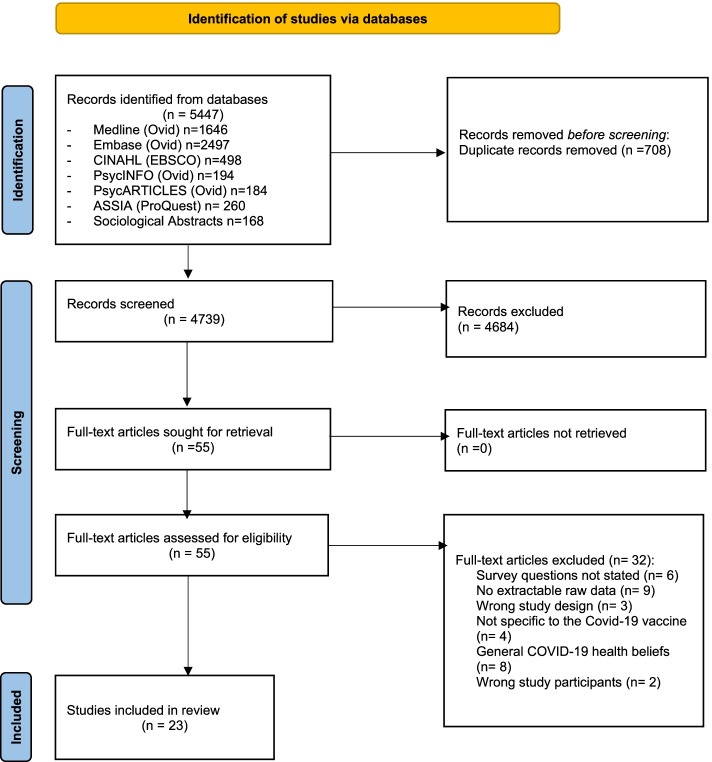
PRISMA Flow Diagram illustrating the summary of search strategy results from initial search to included studies [32]

The literature search returned 5447 studies and following removal of duplicates, 4739 studies were considered for title and abstract screening. Following title and abstract screening, 55 full-text articles were assessed for eligibility. Of these, 23 studies met the inclusion criteria and were deemed eligible for the review [47–69]. Reasons for exclusion at full-text screening included a lack of specific focus on the COVID-19 vaccine and the absence of extractable raw data (Fig. 1). Cohen’s Kappa was 0.7 at title and abstract screening and 0.8 at full-text screening.

### Summary of included studies

All studies had a cross-sectional study design: 22 used online surveys [47–50, 52–69] and one study used a mixed-methods approach, including both an online survey and semi-structured interviews [51]. Eighteen surveys were conducted in the first half of 2020 (January-June) [47–50, 52–57, 59–62, 66–69] and five were conducted in the latter half of 2020 (July-December) [49, 58, 63–65]. Fifteen countries were represented in the review, the commonest being the USA (*n* = 4) [54, 58, 62, 63] and the UK (*n* = 4) [51, 60, 65, 67]. In total, 41,403 participants were sampled across all surveys, with sample sizes ranging from 525 [58] to 5677 [48] participants. The majority of participants were aged between 25 and 50 years old, and all surveys reported a higher proportion of female participants with one exception [58]. Ethnicity data were only reported in nine studies [51, 54, 58, 60–63, 65, 68], with Black, Asian and Minority Ethnic (BAME) representation ranging from 3.6% [68] to 36.7% [54] (Additional file 3).

Survey questions used to assess vaccination intentions could largely be categorised into two; 18 studies used neutral questions such as ‘Will you get the coronavirus vaccine when available?’ [47, 50–54, 56–59, 61, 62, 64–68]; five studies used persuasive language that may have potentially influenced self-reported vaccine acceptance, for example ‘If a new vaccine for COVID-19 was released that was proven to be safe and effective, I would get vaccinated immediately’ [48, 49, 60, 63, 69]. Fifteen studies recorded responses using a Likert-scale, adopting variations of the terms ‘Strongly Agree to Disagree’ [47–49, 51, 53, 57, 59, 62, 63, 65–69], seven studies utilised a simple ‘Yes’, ‘No’ and/or ‘Maybe’ response scale [50, 52, 54–56, 60, 61, 64] and one study used a best-fit statement response [58] (Additional file 4).

### Quality assessment and risk of Bias

Of the 23 studies included in the review, 17 studies used piloted, trialled or previously published survey instruments (Additional file 5) [47, 48, 51, 52, 54–65, 68, 69]. Only 11 studies used an adequate sampling frame to achieve a representative sample [49, 50, 54, 55, 58–60, 62, 63, 65, 66] and 10 studies were deemed to use an adequate selection process [51, 54, 55, 59–61, 63, 65, 66]. Of the studies that used adequate sampling frames, eight used existing online research panels [50, 54, 58, 60, 62, 63, 65, 66] (two most common being Qualtrics, *n* = 2 [58, 60] and the AmeriSpeak panel, *n* = 2 [54, 63]). Sixteen studies did not categorise non-responder rates [47, 49, 51–53, 55, 56, 58, 60, 62, 64–68] and 11 non-responder rates raised concerns over non-response bias [48–50, 53, 57, 59, 63, 64, 66, 67, 69]. Non-responder bias could not be determined for six studies due to lack of adequate information [47, 52, 56, 60, 65, 66].

### Vaccine intentions

**Fig. 2:**
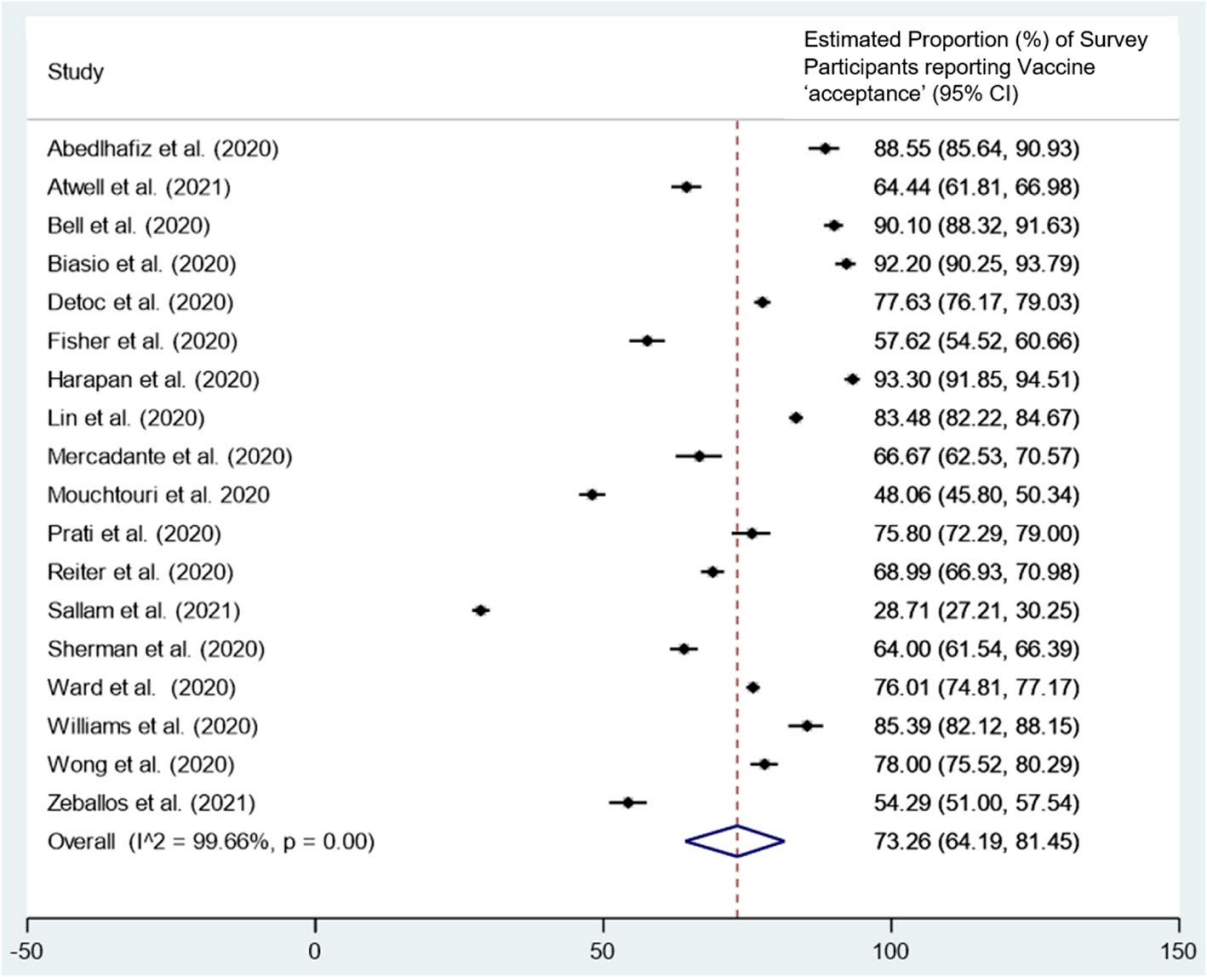
Random-effects meta-analysis of 18 cross-sectional studies [47, 50–54, 56–59, 61, 62, 64–68]: Estimated proportion of survey participants reporting vaccine acceptance (% of total sample). Vaccine ‘acceptance’ is defined as either definite (responding ‘yes/ accepting/ strongly agree/ very likely’) or possible (responding ‘agree/ probably yes/ somewhat likely/ unsure but leaning towards yes’). Vaccine ‘unwillingness’ is defined as hesitant (responding ‘not sure/ neither agree nor disagree/ neutral/ I don’t know/ Maybe/ Hesitant), improbable (responding ‘somewhat unlikely/ disagree/ unsure but leaning towards no/ probably no) or very improbable (responding ‘no/ very unlikely/ strongly disagree/ resistant). CI = confidence interval

Five studies were removed from the meta-analysis due to the use of persuasive questions to assess vaccine intentions [48, 49, 60, 63, 69]. From the 18 studies included in the meta-analysis, the pooled proportion of survey participants willing to receive the COVID-19 vaccine was 73.3% (*n* = 18, 95%CI 64.2 to 81.5%, I^2^ = 99.7%, *p* = 0.00 Fig. 2) [47, 50–54, 56–59, 61, 62, 64–68]. Only two studies included in the meta-analysis reported a higher proportion of participants unwilling to receive the vaccine (71.3% in Sallam et al. [64] and 51.9% in Mouchtouri et al) [59].

Across the 10 studies that included four response categories (variations of ‘strongly agree’, ‘agree’, ‘disagree’ and ‘strongly disagree’), individuals were more confident in accepting the vaccine than rejecting the vaccine [47–49, 51, 53, 57, 59, 62, 67, 68]. A mean proportion of 51.3% participants were definitely willing, compared to only 30.7% participants possibly willing to receive the COVID-19 vaccine. Contrastingly, a higher proportion of participants reported improbable rather than very improbable intentions to receive the COVID-19 vaccine, a mean proportion of 6.0 and 4.9% respectively.

Across the 15 studies that included a hesitant response choice (variations of ‘maybe’), participants were more likely to be vaccine hesitant than either improbable/very improbable, with a mean proportion of 22.2 and 9.4% respectively [47–49, 53, 54, 59–63, 65, 67–69].

### Factors associated with Vaccine intentions

#### Health beliefs

A lower perceived individual risk and perceived severity of COVID-19, lower levels of worry regarding the pandemic and lower perceived likelihood of becoming infected with COVID-19 were all found to be major variables reducing vaccine acceptance in all eight studies investigating these factors [50, 53, 57, 61, 62, 66, 68, 69]. One survey reported that personal fear about COVID-19 meant the individual was almost 2.5 times significantly more likely to accept the vaccine (Odds Ratio (OR) 2.5, 95%CI 2.0-3.0, *p* < 0.001) compared to individuals with no fear [53]. Additionally, positive attitudes towards past influenza vaccines significantly increased the likelihood of COVID-19 vaccine acceptance [50, 54, 65]. Higher levels of perceived vaccine harm, concerns about side-effects and vaccine efficacy significantly contributed to a reduced vaccine acceptance in four out of four studies [57, 62, 65, 68]. One survey reported a significant increase in the likelihood of vaccine acceptances if individuals perceived the vaccine to reduce the risk of COVID-19 infection (OR 3.1, 95%CI 2.1 to 4.8, *p* < 0.001) [57].

#### Sociodemographic variables

##### Sex

**Fig. 3:**
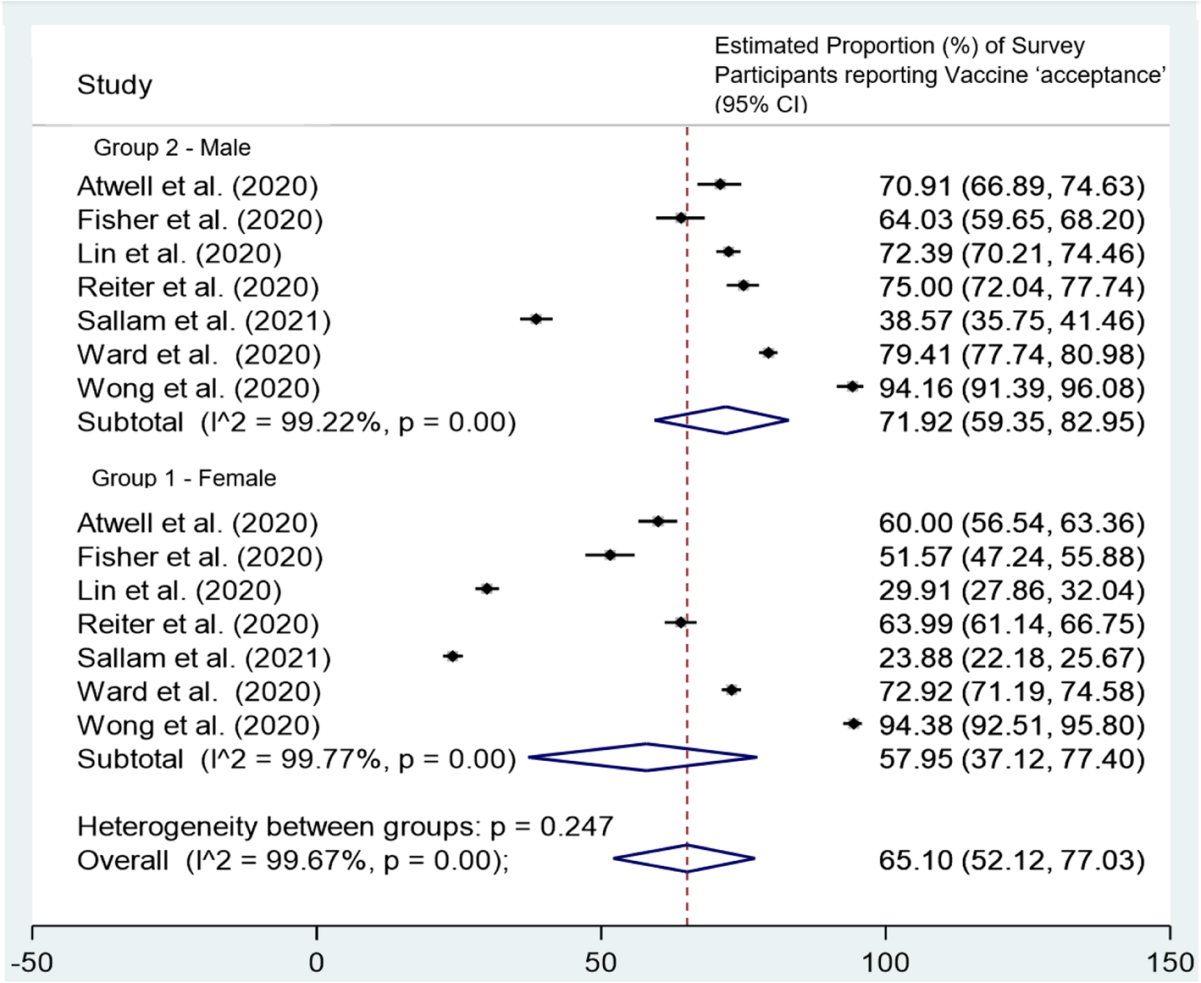
Subgroup analysis by gender of 7 studies [50, 54, 57, 62, 64, 66, 68]: Estimated proportion of survey participants reporting COVID-19 vaccine acceptance (% of total sample in each gender group). Group 1 = Female Participants, Group 2 = Male Participants, CI = confidence interval. Seven studies presented proportions of total survey participants willing to receive the COVID-19 vaccine by gender and hence data was collated in a subgroup analysis. Response proportions were combined into 2 categories; vaccine ‘acceptance’ (responding with either strongly agree or agree to receiving the COVID-19 vaccine) and vaccine ‘unwillingness’ (responding with either maybe, disagree, strongly disagree to receiving the COVID-19 vaccine)

Males were significantly more willing to receive the COVID-19 vaccine than females in all seven studies investigating this variable [50, 54, 57, 62, 64, 66, 68]. One survey reported that males were almost twice as likely as females to receive the COVID-19 vaccine (OR 1.9, 95%CI 1.5-2.3, *p* < 0.001) [53]. A subgroup analysis by gender across the seven studies reporting gender proportions revealed a similar trend; the pooled proportion willing to vaccinate for males was 71.9% (95%CI 59.4 to 83.0%) and 58.0% (95%CI 37.1 to 77.4%) for females, but this was not statistically significant (*p* = 0.247, Fig. 3) [50, 54, 57, 62, 64, 66, 68]. Similarly, females were consistently recorded as more likely to be vaccine hesitant than their male counterparts [49, 50, 60] with an Australian survey recording females as almost twice as likely to be vaccine hesitant than males (Relative Risk Ratio (RRR) = 2.0, 95%CI 1.5 to 2.6, p < 0.001) [50].

##### Ethnicity

BAME individuals reported lower vaccination intentions than White individuals in all four studies that assessed acceptance by ethnicity [51, 54, 60, 62]. Specifically, individuals of Black ethnicity were reported to be less accepting than White ethnic individuals in both studies investigating specific ethnicities [54, 62] and less accepting than both Hispanic and White ethnic individuals in one survey [62]. One study reported Black individuals to be up to 6.4 times more likely to be either hesitant or resistant (RRR 6.4, 95%CI 3.2 to 13.0, no *p*-value reported) than their White counterparts [54].

##### Household income

Individuals with a lower household income were significantly less willing to receive the vaccine in three out of four studies [51, 58, 62]. One study reported that lower income households were over two times more likely to reject the vaccine than higher income households (OR 2.1, 95%CI 1.3-3.3, *p* < 0.001) [51]. However, one survey appeared to contradict this trend, suggesting that individuals in the lowest income band were significantly more likely to express vaccine hesitancy than rejection compared to individuals in higher income brackets [60].

##### Educational attainment

In all three studies investigating education, lower education was associated with lower vaccine acceptance [49, 54, 64]. In one study, the risk of individuals with no high school diploma rejecting and/or hesitating over the vaccine was almost eight times higher than those with a diploma or higher (RRR 7.8, 95%CI 3.1 to 19.6, no *p*-value reported) [54].

##### Age

Four out of seven studies reported younger individuals to be less vaccine willing [53, 58, 65, 66]. However, there were substantial variations in the age groupings used by included studies. Two studies reported individuals aged < 30 years [53] and < 35 years old [66] to be the least willing age group to receive the vaccine (OR 1.5, 95%CI 1.3-1.9, *p* < 0.001 and OR 1.2, 95%CI 1.1-1.5, *p* < 0.001 respectively). One study opposed this trend, reporting individuals aged 35-44 years as most likely to reject (OR 3.3, 95%CI 1.2-9.5, *p* < 0.05) [60]. Another conflicting study drew conclusions from proportions alone, suggesting individuals aged < 35 years old were more vaccine willing than those in older age groups [48].

#### Time of survey

**Fig. 4:**
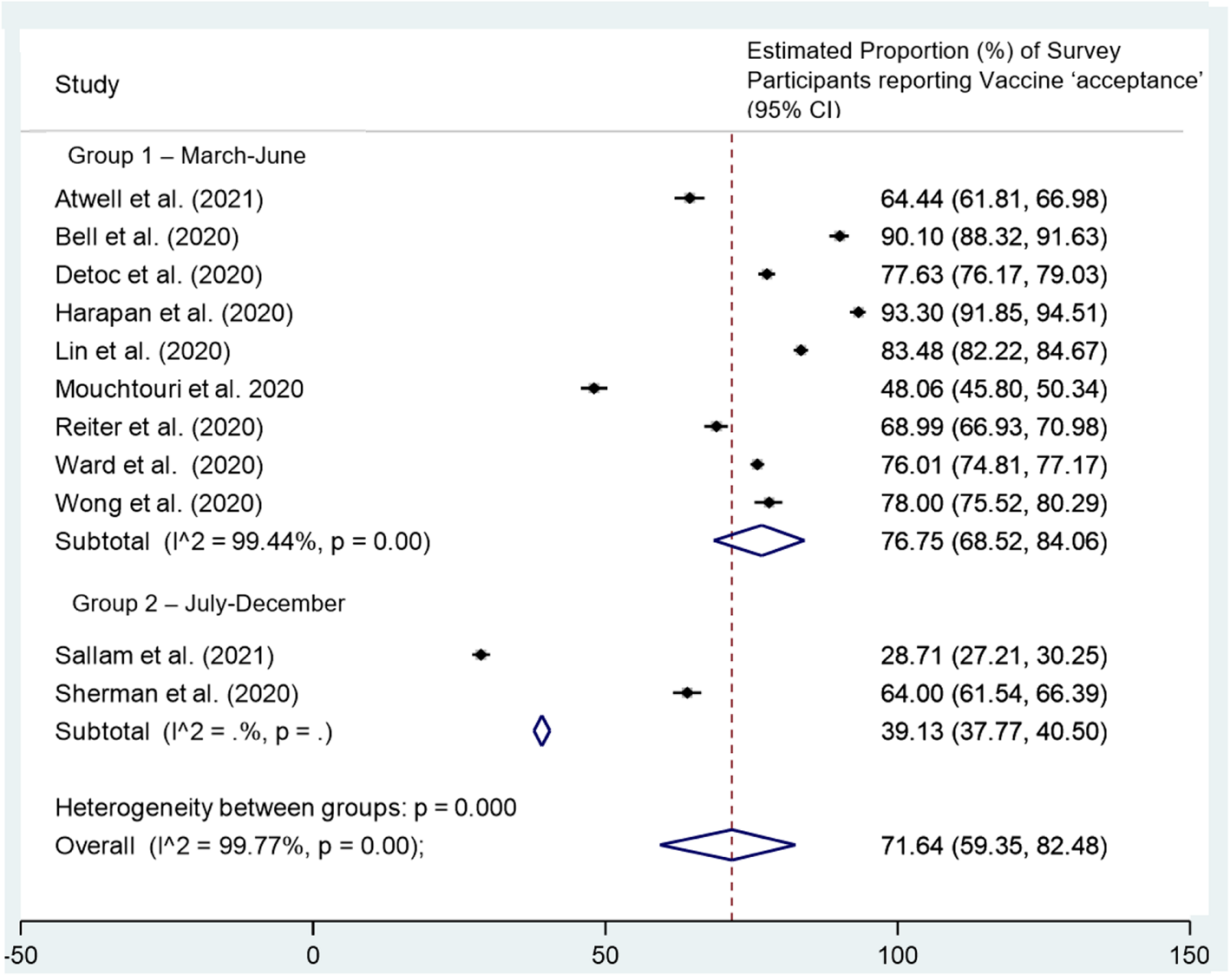
Subgroup analysis by time of survey of 11 studies [50, 51, 53, 56, 57, 59, 62, 64–66, 68]: Estimated proportion of survey participants reporting COVID-19 vaccine acceptance (% of total sample). Only the 11 studies with a sample size ≥1000 were included in the analysis. Group 1 = March-June 2020, Group 2 = July-December 2020, CI = Confidence Interval

Across the 11 studies adopting a large sample size (*n* ≥ 1000), the proportion reporting vaccine acceptance reduced significantly over time [50, 51, 53, 56, 57, 59, 62, 64–66, 68]. The nine surveys conducted between March-June had a pooled mean proportion of 76.8% survey participants reporting vaccine acceptance (*n* = 9, 95%CI 68.5 to 84.1%, *p* = 0.0) [50, 51, 53, 56, 57, 59, 62, 66, 68] compared to 39.1% survey participants reporting vaccine acceptance (*n* = 2, 95%CI 39.1 to 40.5%, p = 0.0) across the two studies conducted between July-December (Fig. 4) [64, 65]. Smaller studies (*n* < 1000) were more likely to increase the heterogeneity of the results, so following the example of Robinson et al. [23], the authors chose to restrict the subgroup analysis to larger studies which may have had more robust estimates of vaccine acceptance.

#### Sampling type

**Fig. 5:**
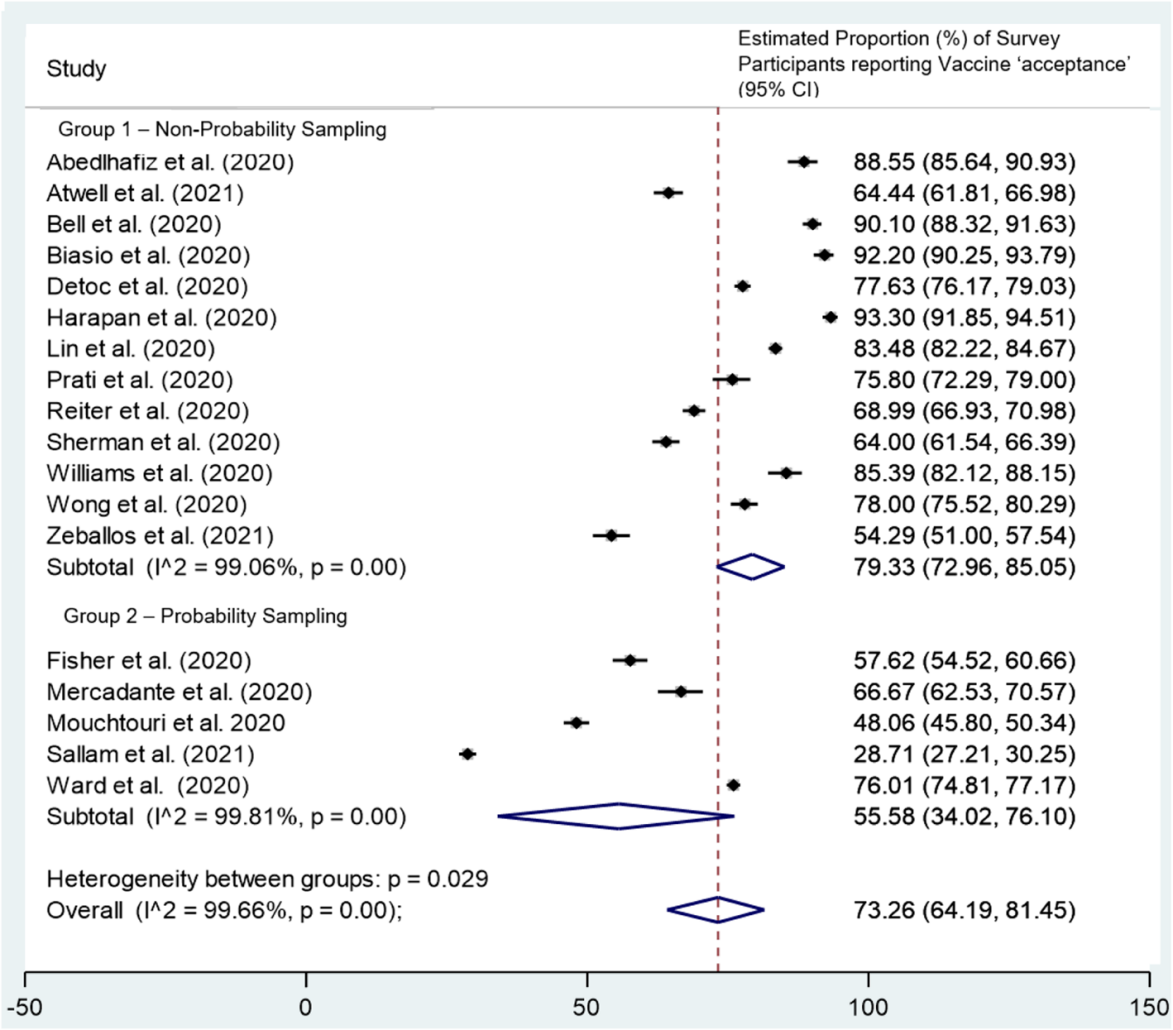
Subgroup analysis of vaccine acceptance by method of recruitment of 18 studies [47, 50–54, 56–59, 61, 62, 64–68]: (ES) estimated proportion of total survey participants reporting COVID-19 vaccine acceptance (% of total sample). 1 = Non-Probability Sampling, 2 = Probability Sampling, CI = Confidence Interval

A subgroup analysis assessing study methodology used for recruitment reveals that survey participants recruited via probability sampling [54, 58, 59, 64, 66] were significantly less willing to receive the COVID-19 vaccine than survey participants recruited via non-probability sampling [47, 50–53, 56, 57, 61, 62, 65, 67–69] (*p* = 0.029, Fig. 5), 55.6% (95%CI 34.0 to 76.1%) compared to 79.3% (95%CI 73.0 to 85.1%) respectively.

### Reasons for Vaccine hesitancy

Concern over vaccine safety was the most common reason reported for both vaccine hesitancy and rejection cited in all six studies investigating vaccine reasoning [51, 54, 57, 58, 62, 67]. Three studies explicitly stated that fears of potential side-effects were the main cause for concern [57, 58, 62]. Other reasons include concern over vaccine efficacy [57, 62], speed of vaccine production and lack of evidence [51, 66], a lack of trust in both scientific and governmental bodies [69], and general anti-vaccination attitudes [51, 66]. For all studies investigating reasons for vaccine willingness, the main justification for vaccine acceptance was for the protection of both the individual and others [51, 57, 67].

## Discussion

### Comparison to existing literature

Guidance on how to achieve high vaccine uptake could be based on existing evidence regarding uptake of previous vaccines and specific research on COVID-19 vaccine intention. This systematic review investigated intentions to receive the COVID-19 vaccination across the global population and the relevant influencing factors, as reported in eligible international cross-sectional studies published between March and December 2020. A total of 4739 journal articles were screened and 23 cross-sectional surveys were selected for inclusion in the review [47–69]. Twenty-one out of the twenty-three studies reported high intentions to receive the vaccine across survey participants [47–58, 60–63, 65–69], with a significant trend towards a declining willingness to vaccinate over time; concordant with the findings of existing reviews investigating COVID-19 vaccine willingness [22–26]. Consistent with similar reviews, the main reasons behind COVID-19 vaccine acceptance reported in this review are for the protection of oneself and others, suggesting that receiving the vaccine is regarded as a social responsibility [22–26].

Compared to self-reported acceptance to receive past-vaccines, the rate of COVID-19 vaccine acceptance was generally higher [70–72]. It is important to note the significant effect of sampling bias on self-reported vaccine acceptance in surveys included in the review. The inclusion of cross-sectional studies that used non-probability recruitment methods may have further limited the generalisability of the review findings to the general population.

This review confirms the majority of findings reflected in existing literature investigating the sociodemographic trends in COVID-19 vaccine acceptance with higher educational attainment and household income, older age and being of White ethnicity to be associated with a higher acceptance [22–26]. Furthermore, this pattern is consistent with literature investigating attitudes towards past-vaccines [73–76]. Literature investigating past-vaccines consistently reports that females are likely to express higher vaccine acceptance in general compared to males [77, 78]. However, the findings of this review investigating attitudes towards the COVID-19 vaccine report the opposite. Research has suggested that females are more likely to lower their intention to vaccinate following exposure to vaccine misinformation than males. The widespread conspiracy that the COVID-19 vaccine causes infertility may have contributed to this significant difference [79, 80]. This same misconception posed an obstacle to uptake of the Polio vaccine in Nigeria, India and Pakistan [80]. Females are more likely to express greater levels of concern towards their personal health than males which could explain why females are more inclined to believe COVID-19 vaccine conspiracies [81]. Social media is reported to be the main source of vaccine misinformation [82]. The narrative of social media is rapidly changing, suggesting that the influence of social media on vaccine intentions is subject to change. The findings of this review are supported by two recent systematic reviews conducted in 2021 [22, 23]. Interestingly, Lin et al.’s narrative review reported inconsistent findings towards the influence of sex on COVID-19 vaccine acceptance [22]. Similarly, Robinson et al. stated that whilst seven out of fourteen studies reported that females have significantly lower COVID-19 vaccine intentions than males, five studies reported no significant association between COVID-19 vaccine acceptance and sex [23].

The association between household income and willingness to receive the COVID-19 vaccine has been echoed throughout the literature [22–26, 73–76]. Unwillingness to receive the COVID-19 vaccine can be described as a negative health behaviour. In the literature, there is an established correlation between low income level and negative health behaviours, which may provide an explanation for the association between income and vaccine willingness [83]. However, the included studies did not control for confounding factors that may have influenced the strength of the association between household income and vaccine willingness including education level and health literacy; ethnicity; access to vaccines and healthcare services; and urban vs rural living to name a few [84–87]. A Finnish study suggests it may be more appropriate to use the term ‘low socio-economic status’ rather than ‘low household income’ to account for the confounding variables that may be contributing to this association [88, 89]. Going forward, it would be interesting to further examine the impact that these confounding variables may have had on the association between income and willingness. This may identify areas to target in an attempt to encourage vaccine uptake in this population group.

A recent study published in January 2022 echoed the findings of this review, discovering that COVID-19 vaccine acceptance was lower amongst minority groups and less educated individuals [90]. Another study discovered that 48% of unvaccinated African-Americans were reported to be vaccine-hesitant, and of such individuals, rates of hesitancy were highest among the lesser educated [21]. These disparities may be due to concerns regarding the side-effect profile of the vaccine and found a higher acceptance for a combination vaccine (COVID-19 and influenza) than for COVID-19 alone among minorities [90]. This suggests that novelty of the vaccine may be contributing to the risk-profile, highlighting areas that need to be addressed to combat low vaccine uptake among minority groups.

Risk perception is a well-established determinant in vaccine decision-making [91, 92]. This pattern of behaviour is reported as being no different for the COVID-19 vaccine and can be explained by the Health Belief Model; individuals are more likely to engage in health-protective behaviours if they perceive themselves to be at a higher risk from the disease in question [93, 94]. The high level of uncertainty towards the threat of COVID-19 and rapid rate of transmission of the virus substantially increased individuals’ perceived risk of ill-health and state of anxiety during the pandemic, motivating individuals to perform health-protective behaviours [95–97].

### Further research

This review has contributed to the literature in providing the most recent representation of the public’s views towards the COVID-19 vaccine around the globe. The findings of this review suggest that global policymakers cannot rely on the findings of existing literature about past-vaccines to formulate public health campaigns regarding COVID-19 vaccine uptake. Despite similar social pressures and the influence of risk perception on the uptake of both existing vaccines and the COVID-19 vaccine, there are several sociodemographic aspects specific to the COVID-19 vaccine that need to be considered and further researched, particularly in terms of sex and age. Public attitudes towards the COVID-19 vaccine around the globe need to be continuously explored as there appears to be evidence that attitudes can change rapidly, for example, the influence of social media on differences in vaccine acceptance between males and females. Consequently, we cannot rely solely on existing findings of past-vaccines and early COVID-19 vaccine research to guide government advice.

Since the rollout of COVID-19 vaccine programmes, booster doses of the vaccine have emerged, as the duration of protection provided by the vaccine is currently unknown [98, 99]. A cross-sectional study among the American population suggested a strong predictor of booster hesitancy is primary COVID-19 vaccine status [30]. The sociodemographic trends in vaccine hesitancy towards booster doses must be investigated, as it is currently unclear whether the sociodemographic trends in vaccine hesitancy towards primary doses of the COVID-19 vaccine that were highlighted in this review, can be directly applicable to further booster doses. If there are other influencing factors at play, these must be identified and closely monitored by public health officials to guarantee the success of the vaccine and achieve herd immunity.

### Translation into practice

Following the subsequent roll-out of mass-vaccination programmes across the globe, uptake of the COVID-19 vaccine has been higher than anticipated. As of August 2021, Israel has successfully vaccinated over 70% of all adults over the age of 16 [100] and the UK has almost achieved 80% of all adults over the age of 16 double vaccinated [101]. The literature suggests there are considerable discrepancies between decision-making in real-life and hypothetical situations, with individuals more likely to focus on the outcome of decisions in real-life situations [102]. As evidenced in this review, a major reason behind an individual’s intention to receive the COVID-19 vaccine may be the protection of others as well as themselves. This suggests that receiving the vaccine may be seen as a social responsibility [103]. The UK no longer recommends the use of AstraZeneca in under-40s with no underlying health conditions following reports that the AstraZeneca vaccine may have a higher risk of blood clots than other vaccines [104–106]. However, this association was later disproved following a review by the European Medicines Agency [107]. Nevertheless, the Pfizer vaccine is the only vaccine authorised for young adults aged 12-17 years old in the UK [108] and numerous European countries have stopped administering the AstraZeneca vaccine across all age-groups [109]. It is important to acknowledge the political implications that Brexit may have had on the decision of the European Union to discontinue the use of AstraZeneca, a UK-made vaccine [110, 111]. This decision may have fuelled vaccine hesitancy, with several European polls reporting a substantial drop in perceived vaccine safety following the AstraZeneca blood clot scares [110]. Following the development of several licenced vaccines, vaccine acceptability and personal risk perceptions may be further affected by the type of vaccine offered to individuals. Thus, the reporting of vaccine risk assessments must be carefully navigated and the prevention of vaccine misinformation across social media is imperative if a high vaccine uptake is to be achieved across the globe.

This review has identified sub-groups of the population that are at a higher risk of vaccine hesitancy and low vaccine uptake. There is therefore a continued risk of pockets of local outbreaks across sub-groups of the population despite the vaccine now being available [112]. The findings of this review can guide local policy-makers towards the close monitoring of vaccine uptake amongst sub-groups of the population at risk of vaccine hesitancy, now vaccines are available. It is also important to acknowledge the impact that COVID-19 vaccine accessibility may have had on vaccine uptake, especially in low-economically developed countries where there are well-documented issues pertaining to vaccine inequity [113, 114]. This review has highlighted the dangerous impact that vaccine misinformation can have on vaccine hesitancy, and thus can be used by local policy-makers to control the spread of COVID-19 misinformation on social media, focusing particularly on debunking COVID-19 vaccine myths targeted towards individuals at a higher risk of vaccine hesitancy. This review contributes to the growing evidence base suggesting that males are more likely to receive the COVID-19 vaccine than females [24, 25], opposing the trend in past-vaccine hesitancy across the sexes reported in existing literature [71, 72].

### Strengths and limitations

This systematic review has several strengths. Multiple databases were searched and there was a high level of agreement between screeners. Thorough quality and risk of bias assessments were also undertaken using validated tools that were piloted before use. However, searches were limited to English language studies and grey literature was explicitly excluded to ensure a manageable volume of literature was retrieved. This may have led to the exclusion of relevant literature. In a similar vein, we acknowledge that we were unable to search every possible database and that by omitting searches of databases such as Web of Science and Scopus, we may have missed a small number of potentially eligible studies that were only indexed in those databases.

It is important to acknowledge the fast-moving nature of the COVID-19 pandemic; four licenced vaccines are now available whereas there were either no/limited vaccines available at the time when eligible studies were conducted [115]. The availability of vaccines may have a role in vaccine intentions. Due to the nature of systematic reviews, we were only able to focus on factors contributing to vaccine hesitancy that were cited within the papers eligible for inclusion in the review. It is important to acknowledge that multiple factors may contribute to vaccine hesitancy and this review does not provide an exhaustive list, there may be other factors at play that contribute to both vaccine hesitancy and willingness. We were unable to explore the impact of issues such as access to the COVID-19 vaccine as this was outside the scope of the review, but it is nevertheless important to acknowledge.

## Conclusion

Overall, the review discovered positive attitudes towards the COVID-19 vaccine before February 2021, with 73% of the total survey participants reporting a high intention to receive the COVID-19 vaccine. COVID-19 vaccine acceptance can be influenced by many sociodemographic factors and individual risk perception towards COVID-19. The findings of this review imply that future research should explore the reasoning behind vaccine intentions for different sociodemographic groups, to allow targeted communication strategies to be formulated by governments and public health agencies. The impact of both vaccine availability and reported adverse effects must be monitored so public health policies can address these concerns. A high vaccine uptake to current mass-vaccination programmes and potential booster vaccinations is essential to achieve the end goal of herd immunity and combat any potential future variants.

## Supplementary Information

**Additional file 1. **Eligibility Criteria. Eligibility criteria for the research question, using the SPIDER search tool.

**Additional file 2.** Search Strategy. A detailed description of the search strategies for each database searched in the review.

**Additional file 3.** Study Characteristics Table. Summary table detailing the characteristics of each included cross-sectional study.

**Additional file 4.** Study Results Table. Summary table detailing the results of each included cross-sectional study.

**Additional file 5.** AXIS Summary Table [41]. Summary table of results of the appraisal of cross-sectional studies (AXIS) for each study.

**Additional file 6.** Data Extraction Form. A copy of the data extraction form used to extract data from each study included in the review.

**Additional file 7.** AXIS Form. A copy of the AXIS form used for the assessment of bias and quality of each study included in the review.

## Data Availability

The datasets used and/or analysed during the current study available from the corresponding author on reasonable request.

## Abbreviations

COVID-19: Coronavirus Disease 2019
WHO: World Health Organisation
UK: United Kingdom
DTP: Diphtheria-tetanus-pertussis Vaccine
A/H1N1: Influenza A Virus subtype H1N1
PHE: Public Health England
CI: Confidence Interval
SPIDER: Sample, Phenomenon of Interest, Design, Evaluation, Research Type
PICO: Population, Intervention, Comparison, Outcomes
AXIS: Appraisal Tool for Cross-Sectional Studies
USA: United States of America
BAME: Black, Asian and minority ethnic
OR: Odds Ratio
RRR: Relative Risk Ratio
NHS: National Health System
