# Supplementary material for: Factors associated with COVID-19 vaccine intentions during the COVID-19 pandemic; a systematic review and meta-analysis of cross-sectional studies
Source: BMC Public Health. 2022 Sep 2;22:1667. doi: 10.1186/s12889-022-14029-4 (PMC9437387; doi:10.1186/s12889-022-14029-4)
Supplement: Supplementary file 1 — Additional file 1. Eligibility Criteria. Eligibility criteria for the research question, using the SPIDER search tool. [file 12889_2022_14029_MOESM1_ESM.docx]

**Additional File 1: Eligibility Criteria**

| **SPIDER** | **EXPLANATION** |
| --- | --- |
| **Sample** | Adults from the general population.  Studies were excluded if limited to healthcare professionals and medical students only as this would have restricted the generalisability of the review findings to the general population. |
| **Phenomenon of Interest** | Beliefs about the COVID-19 vaccine including, but not limited to, vaccine hesitancy or acceptance, factors influencing acceptance and reasoning behind vaccine intentions. |
| **Design** | Cross-sectional studies based on survey data. |
| **Evaluation** | Surveys must have reported some metric relating to COVID-19 vaccine intentions and/or respective determinants and/or reasoning.  This includes any type of COVID-19 vaccine.  This must have been presented as extractable raw data.  Where sociodemographic factors were reported, data on these were extracted for analysis.  Studies must have detailed the specific survey questions used to assess attitudes, to allow for appropriate analysis. |
| **Research Type** | Studies published in peer-reviewed journals were eligible for inclusion.  Unpublished journal articles were not included due to the low quality of data they were likely to provide, affecting the validity of the analysis and discussion. Grey literature was not included, as due to the high level of misinformation surrounding the subject of COVID-19, it would have been inappropriate to include this datatype in the evidence base of the systematic review. This did not limit the generalisability of the literature search, as the initial scoping searches retrieved 19 cross-sectional studies, published in peer-reviewed journals. Editorials, reviews, or commentaries were excluded. Dissertations and theses were not considered as there would have been no literature available in this evidence area due to the recent emergence of COVID-19. |

**Table 1.** *Eligibility criteria for the research question, using the SPIDER search tool.*
