# Supplementary material for: Factors associated with COVID-19 vaccine intentions during the COVID-19 pandemic; a systematic review and meta-analysis of cross-sectional studies
Source: BMC Public Health. 2022 Sep 2;22:1667. doi: 10.1186/s12889-022-14029-4 (PMC9437387; doi:10.1186/s12889-022-14029-4)
Supplement: Supplementary file 2 — Additional file 2. Search Strategy. A detailed description of the search strategies for each database searched in the review. [file 12889_2022_14029_MOESM2_ESM.docx]

**Additional File 2: Search Strategy**. A detailed description of the search strategies for each database searched in the review.

Ovid Interface: Medline and In-Process & Other Non-indexed Citations^44^

Search Date: 12/02/2021

| **Search Line** | **Search Terms** | **Results** |
| --- | --- | --- |
| **1** | (“COVID-19” [MeSH Terms] or "2019 Novel Coronavirus Disease" or "2019 Novel Coronavirus Infection" or "2019-nCoV Disease" or "2019-nCoV Infection" or "COVID-19 Pandemic" or "COVID-19 Pandemics" or "COVID-19 Virus Disease" or "COVID-19 Virus Infection" or "COVID19" or "Coronavirus Disease 2019" or "Coronavirus Disease-19" or "SARS Coronavirus 2 Infection" or "SARS-CoV-2 Infection").mp | 79402 |
| **2** | exp Pandemics/ [MeSH Heading] | 4990 |
| **3** | exp Intention/ [MeSH Heading] | 12036 |
| **4** | exp Attitude to Health/ [MeSH Heading] | 428488 |
| **5** | exp Culture/ [MeSH Heading] | 163750 |
| **6** | exp Trust/ [MeSH Heading] | 10155 |
| **7** | exp Psychology/ [MeSH Heading] | 67767 |
| **8** | exp Social Control Policies/ [MeSH Heading] | 156408 |
| **9** | (“prevention and control” OR “prevention & control”).mp | 1332083 |
| **10** | Public Health [Mesh Heading] | 8073149 |
| **11** | exp “Mass Vaccination”/ [MeSH Heading] OR exp “Vaccination Refusal”/ [MeSH Heading] OR exp “Anti-Vaccination Movement”/ [MeSH Heading] OR exp “Vaccination Coverage”/ [MeSH Heading] OR exp “Vaccination”/ [MeSH Heading] | 87422 |
| **12** | 1 OR 2 | 80370 |
| **13** | 3 OR 4 OR 5 OR 6 OR 7 | 649904 |
| **14** | 8 OR 9 OR 10 OR 11 | 8730744 |
| **15** | 12 AND 13 AND 14 | 1727 |
| **16** | Limit 15 to (English language and yr=”2020-Current”) | 1646 |

Ovid interface: Embase^45^

Search Date: 12/02/2021

| **Search Line** | **Search Terms** | **Results** |
| --- | --- | --- |
| 1 | ("2019 Novel Coronavirus Disease" or "2019 Novel Coronavirus Infection" or "2019-nCoV Disease" or "2019-nCoV Infection" or "COVID-19 Pandemic" or "COVID-19 Pandemics" or "COVID-19 Virus Disease" or "COVID-19 Virus Infection" or "COVID19" or "Coronavirus Disease 2019" or "Coronavirus Disease-19" or "SARS Coronavirus 2 Infection" or "SARS-CoV-2 Infection").mp. | 90842 |
| 2 | COVID-19.mp. | 86674 |
| 3 | exp pandemic/ | 54585 |
| 4 | exp motivation/ | 106935 |
| 5 | exp attitude to health/ | 115950 |
| 6 | (cultur* or “cultur* background*” or “cultur* relativism*” or belief*”).mp. | 2046607 |
| 7 | exp trust/ | 25147 |
| 8 | exp psychology/ | 355534 |
| 9 | (“social control polic*” or “control polic* social”).mp. | 22 |
| 10 | exp “prevention and control”/ | 2157629 |
| 11 | exp public health/ | 188286 |
| 12 | exp vaccination refusal/ or exp vaccination/ or exp anti-vaccination movement/ or exp vaccination coverage/ | 174300 |
| 13 | exp mass-immunization/ | 3607 |
| 14 | exp social control/ | 347577 |
| 15 | 1 OR 2 OR 3 | 111037 |
| 16 | 4 OR 5 OR 6 OR 7 OR 8 | 2544964 |
| 17 | 9 OR 10 OR 11 OR 12 OR 13 OR 14 | 2618056 |
| 18 | 15 AND 16 AND 17 | 2938 |
| 19 | Limit 18 to (English language and yr=”2020-Current”) | 2497 |

Ovid Interface: APA PsycINFO^47^

Search date: 12/02/2021

| **Search Line** | **Search Terms** | **Results** |
| --- | --- | --- |
| 1 | ("2019 Novel Coronavirus Disease" or "2019 Novel Coronavirus Infection" or "2019-nCoV Disease" or "2019-nCoV Infection" or "COVID-19 Pandemic" or "COVID-19 Pandemics" or "COVID-19 Virus Disease" or "COVID-19 Virus Infection" or "COVID19" or "Coronavirus Disease 2019" or "Coronavirus Disease-19" or "SARS Coronavirus 2 Infection" or "SARS-CoV-2 Infection").mp. | 3586 |
| 2 | COVID-19.mp. | 2225 |
| 3 | exp Pandemics/ | 1865 |
| 4 | exp Intention/ | 17460 |
| 5 | exp Health Attitudes/ | 10686 |
| 6 | (cultur* or “cultur* background” or “cultur* relativism*”).mp. | 349226 |
| 7 | exp “Trust (Social Behavior)”/ | 11518 |
| 8 | exp Psychology/ | 198957 |
| 9 | exp Social Control/ | 7768 |
| 10 | (“social control polic*” or “control polic*, social”).mp. | 174 |
| 11 | “prevention and control”.mp. | 1213 |
| 12 | exp Public Health/ | 33230 |
| 13 | exp Immunization/ | 4764 |
| 14 | (vaccination or “vaccin* coverage*” or “vaccin* refusal*” or “anti-vaccin* movement” or “mass-immunization”).mp. | 4553 |
| 15 | 1 OR 2 OR 3 | 4151 |
| 16 | 4 OR 5 OR 6 OR 7 OR 9 | 558533 |
| 17 | 9 OR 10 OR 11 OR 12 OR 13 OR 14 | 47412 |
| 18 | 15 OR 16 OR 17 | 290 |
| 19 | limit 18 to (english language and yr=”2020-Current”) | 194 |

Ovid Interface: APA PsycARTICLES^48^

Search Date: 12/02/2021

| **Search Line** | **Search Terms** | **Results** |
| --- | --- | --- |
| 1 | ("2019 Novel Coronavirus Disease" or "2019 Novel Coronavirus Infection" or "2019-nCoV Disease" or "2019-nCoV Infection" or "COVID-19 Pandemic" or "COVID-19 Pandemics" or "COVID-19 Virus Disease" or "COVID-19 Virus Infection" or "COVID19" or "Coronavirus Disease 2019" or "Coronavirus Disease-19" or "SARS Coronavirus 2 Infection" or "SARS-CoV-2 Infection").mp. | 403 |
| 2 | COVID-19.mp. | 312 |
| 3 | pandemic*.mp. | 556 |
| 4 | (intention* or motivation* or disincentive* or expectation*).mp. | 78577 |
| 5 | (“attitude* to health” or “health attitude” or “treatment adherence and compliance” or “therapeutic adherence” or “therapeutic compliance” or “treatment adherence” or “treatment compliance” or “therapeutic adherence and compliance” or “health belief” or belief* or “health belief model” or “health-related belief*” or “health risk behavior*” or “health-protective behavior*” or “health-protective behavior*” or “health protection” or “preventative measures” or “health-preventative measures” or “health-prevention behavior*” or prevention*).mp. | 52633 |
| 6 | (cultur* or “cultur* relativism*” | 62595 |
| 7 | trust*.mp. | 18809 |
| 8 | psychol*.mp. | 213108 |
| 9 | confidence.mp. | 35381 |
| 10 | (“social control polic*” or “control polic*, social”).mp. | 5 |
| 11 | (“prevention and control” or “prevention & control”).mp. | 323 |
| 12 | (vaccination or “vaccination refusal” or “vaccination coverage” or “anti-vaccination movement” or “mass-vaccination” or “mass-immunization”).mp. | 305 |
| 13 | (“public health” or “community health”).mp. | 10428 |
| 14 | 1 OR 2 OR 3 | 596 |
| 15 | 4 OR 5 OR 6 OR 7 OR 8 OR 9 | 215649 |
| 16 | 10 OR 11 OR 12 OR 13 | 10801 |
| 17 | 14 OR 15 OR 16 | 252 |
| 18 | Limit 17 to yr=”2020- Current” | 184 |

EBSCO Interface: CINAHL^46^

Search Date: 12/02/2021

| **Search Line** | **Search Terms** | **Results** |
| --- | --- | --- |
| 1 | (MM“COVID-19”) OR (MH“Coronavirus Infections+”) OR ("2019 Novel Coronavirus Disease" or "2019 Novel Coronavirus Infection" or "2019-nCoV Disease" or "2019-nCoV Infection" or "COVID-19 Pandemic" or "COVID-19 Pandemics" or "COVID-19 Virus Disease" or "COVID-19 Virus Infection" or "COVID19" or "Coronavirus Disease 2019" or "Coronavirus Disease-19" or "SARS Coronavirus 2 Infection" or "SARS-CoV-2 Infection") OR COVID-19 OR pandemic* | 24585 |
| 2 | (MM”Intention”) | 4057 |
| 3 | (MH”Attitude to Health+”) | 162951 |
| 4 | (MH”Culture+”) | 127790 |
| 5 | (MM”Trust”) | 3485 |
| 6 | (MH”Psychology+”) | 25512 |
| 7 | (MH”Social Control+”) | 426502 |
| 8 | “prevention and control” OR “prevention & control” | 611114 |
| 9 | (MH”Vaccines+”) | 47831 |
| 10 | (MM”Vaccination Coverage”) | 252 |
| 11 | “vaccination refusal” OR “mass-vaccination” OR “mass-immunization” | 13623 |
| 12 | (MM”Anti-Vaccination Movement”) | 62 |
| 13 | (MH”Public Health+”) | 1210587 |
| 14 | 2 OR 3 OR 4 OR 5 OR 6 | 310747 |
| 15 | 7 OR 8 OR 9 OR 10 OR 11 OR 12 OR 13 | 1904207 |
| 16 | 1 OR 14 OR 15 | 569 |
| 17 | Limit 16 to Publication Year: 2020-2021. Narrow by Language: English. Expanders- Apply equivalent subjects. Search modes- Boolean/Phrase | 498 |

Sociological Abstracts^49^

Search Date: 12/02/2021

| **Search Line** | **Search Terms** | **Results** |
| --- | --- | --- |
| **1** | ("2019 Novel Coronavirus Disease" or "2019 Novel Coronavirus Infection" or "2019-nCoV Disease" or "2019-nCoV Infection" or "COVID-19 Pandemic" or "COVID-19 Pandemics" or "COVID-19 Virus Disease" or "COVID-19 Virus Infection" or "COVID19" or "Coronavirus Disease 2019" or "Coronavirus Disease-19" or "SARS Coronavirus 2 Infection" or "SARS-CoV-2 Infection") OR COVID-19 OR pandemic* | 5285 |
| 2 | Intention* OR motivation* OR disincentive* OR incentive* OR expectation | 225844 |
| 3 | “attitude* to health” OR “health attitude*” OR “treatment adherence and compliance” OR “therapeutic adherence” OR “therapeutic compliance” OR “treatment adherence” OR “treatment compliance” OR “therapeutic adherence and compliance” OR “health belief*” OR belief* OR “health belief model” OR “health-related belief*” OR “health knowledge” OR “knowledge and belief*” OR “health risk behaviour*” OR “health-protective behaviour*” OR “health-protective behaviour*” OR “health protection” OR “prevention measures” OR “health-preventative measures” OR “health-prevention behaviour” OR prevention* | 207561 |
| 4 | cultur* OR “cultur* background*” OR “culture* relativism*” OR “belief*” | 617285 |
| 5 | trust* OR confidence | 106197 |
| 6 | psychol* | 235687 |
| 7 | noft(“social control polic*” OR noft(“control polic*, social”) | 13638 |
| 8 | noft(“prevention and control”) OR noft(“prevention & control”) | 3111 |
| 9 | noft(vaccination) OR noft(“vaccination refusal”) OR noft(“vaccination coverage”) OR noft(“anti-vaccination movement”) OR noft(“mass-vaccination”) OR noft(“mass-immunization”) | 1304 |
| 10 | noft(“public health) OR noft(“community health”) | 47861 |
|  | 1 AND (2 OR 3 OR 4 OR 5 OR 6) AND (7 OR 8 OR 9 OR 10) Limit to Publication Year: 2020-2021. Narrow by Language: English. Expanders- Apply equivalent subjects. Search modes- Boolean/Phrase | 168 |

Applied Social Sciences Index and Abstracts^50^

Search Date: 12/02/2021

| **Search Line** | **Search Terms** | **Results** |
| --- | --- | --- |
| 1 | "2019 Novel Coronavirus Disease" or "2019 Novel Coronavirus Infection" or "2019-nCoV Disease" or "2019-nCoV Infection" or "COVID-19 Pandemic" or "COVID-19 Pandemics" or "COVID-19 Virus Disease" or "COVID-19 Virus Infection" or "COVID19" or "Coronavirus Disease 2019" or "Coronavirus Disease-19" or "SARS Coronavirus 2 Infection" or "SARS-CoV-2 Infection" | 1374 |
| 2 | Noft(COVID-19) | 2367 |
| 3 | Noft(pandemic*) | 3043 |
| 4 | Noft(intention*) OR noft(motivation*) OR noft(incentive*) OR noft(disincentive*) OR noft(expectation*) | 72425 |
| 5 | Noft(“attitude* to health”) OR noft(“health attitude*”) OR noft(“treatment adherence and compliance”) OR noft(“therapeutic adherence”) OR noft(“therapeutic compliance”) OR noft(“treatment adherence”) OR noft(“treatment compliance”) OR noft(“therapeutic adherence and compliance”) OR noft(“health belief*” OR belief*) OR noft(“health belief model”) OR noft(“health-related belief*”) OR noft(“health knowledge”) OR noft(“knowledge and belief*”) OR noft(“health risk behaviour*”) OR noft(“health-protective behaviour*”) OR noft(“health-protective behaviour*”) OR noft(“health protection”) OR noft(“prevention measures”) OR noft(“health-preventative measures”) OR noft(“health-prevention behaviour”) OR noft(prevention*) | 98179 |
| 6 | Noft(culture*) OR noft(“cultur* background*”) OR noft(“cultur* relativism*” OR noft(“belief*”) | 124251 |
| 7 | Noft(trust*) OR noft(confidence) | 51349 |
| 8 | Noft(psychol*) | 254335 |
| 9 | Noft(“social control polic*” OR noft(“control polic*, social”) | 213 |
| 10 | Noft(“prevention and control”) OR noft(“prevention & control”) | 7187 |
| 11 | Noft(vaccination) OR noft(“mass-vaccination”) OR noft(“mass-immunization”) OR noft(“vaccination refusal”) OR noft(“vaccination coverage”) OR noft(“anti-vaccination movement”) | 3715 |
| 12 | Noft(“public health”) OR noft(“community health”) | 69663 |
| 13 | (1 OR 2 OR 3) AND (4 OR 5 OR 6 OR 7 OR 8) AND (9 OR 10 OR 11 OR 12) Limit to Publication Year: 2020-2021. Narrow by Language: English. Expanders- Apply equivalent subjects. Search modes- Boolean/Phrase | 260 |
