# Supplementary material for: Factors associated with COVID-19 vaccine intentions during the COVID-19 pandemic; a systematic review and meta-analysis of cross-sectional studies
Source: BMC Public Health. 2022 Sep 2;22:1667. doi: 10.1186/s12889-022-14029-4 (PMC9437387; doi:10.1186/s12889-022-14029-4)
Supplement: Supplementary file 3 — Additional file 3. Study Characteristics Table. Summary table detailing the characteristics of each included cross-sectional study. [file 12889_2022_14029_MOESM3_ESM.docx]

# **Additional File 3: Study Characteristics Table**

| **Author (Year of Publication)**  **Table** *continued…* | **Survey Date/s** | **Study Setting; Recruitment; Sample Size (n)** | **Study Design; Sampling Frame** | **Median Age Category [IQR] or Mean [SD]** | **Number of Female Participants (%)** | **Modal Level of Education; Range of Education Levels / Mean Years of Education [SD]** | **Median Income Level [IQR]; Modal Occupation** | **BAME ethnicity (%)** |
| --- | --- | --- | --- | --- | --- | --- | --- | --- |
| Abdelhafiz et al. (2020)^47^ | March, 2020 | Egypt; convenience sampling; n=559 | Online survey; general population of adults who use social media. | 30-<40 [18-<30 to 40-<50] | 348 (62.3) | University; read and write to higher studies | Monthly income (LE):  >10,000 [5000-<8000 to >10,000] | - |
| Ali et al. (2020)^48^ | 28^th^ March- 4^th^ April 2020 | Bahrain; snowball sampling; n=5677 | Online survey; general population of adults who use social media. | 25-34 [18-24 to 35-44] | 3945 (69.5) | College/higher education; primary school to college/higher education | Does not work/study in health care sector | - |
| Alley et al. (2021)^49^ | 9-19^th^ April, 30^th^ July-16^th^ August 2020 | Australia; convenience sampling; n=575 | Online survey; Australian residents aged 18 years or over who use email, news media, social media networks | 55–64 [55-64 to ≥65] | 299 (69.6) | Bachelor and above; Year 12 or below to Bachelor and above | Weekly household income:  $1000-<$2000 [$1000-<$2000 to ≥$2000] | - |
| Atwell et al. (2021)^50^ | 18^th^ - 29^th^ May 2020 | Australia; stratified convenience sample; n=1316 | Online survey; The Values Project Panel | 58 [13.2] | 790 (60.0) | Mean years of education=13.7 [3.4] | - | - |
| Bell et al. (2020)^51^ | 19^th^ April- 11^th^ May 2020 | UK; convenience sampling; n=1252 | Mixed-methods; parents/guardians in England who used social media or email. | 33.0 [4.6] | 1190 (95.0) | - | Annual household income:  £35,000–£84,999 [£35,000–£84,999 to £85,000 and over]; working full-time (over 30h per week) or on parental leave from full-time employment | 74 (5.9) |
| Biasio et al. (2020)^52^ | 6^th^- 16^th^ June 2020 | Italy; convenience, non-probability sampling; n=885 | Online survey; Italians adults from the mailing list of Giovannii Lorenzini Foundation and/or who use social media | 31–50 [31-50 to 51-65] | 443 (50.1) | Tertiary education; primary education to tertiary education | Employed | - |
| Detoc et al. (2020)^53^ | 26^th^ March – 20^th^ April 2020 | France; n=3259 | Online survey; French adult general population and adult patients who use social media and/or attend medical centres | 30–49 [30-49 to 50-64] | 2196 (67.4) | - | Non-healthcare worker | - |
| Fisher et al. (2020)^54^ | 16^th^ – 20^th^ April 2020 | US; n=991 | Online survey via the AmeriSpeakOmnibus Survey; probability-based research panel | 48 [18.1] | 510 (51.5) | College graduate or above; no high school diploma to college graduate or above | Annual Household Income:  $30,000-<$60,000 [<$30,000 to $60,000 - <$100,000]; Working as a paid employee | 364 (36.7) |
| Garcia et al. (2020)^55^ | 18^th^ April- 5^th^ May 2020 | Chile; snowball and convenience sampling; n=566 | Online questionnaire; active recruitment system | 40-49 [30-39 to 40-49] | 329 (58.1) | University education; basic or technical education to university education | Employee or businessman working remotely | - |
| Harapan et al. (2020)^56^ | 25^th^ March- 6^th^ April 2020 | Indonesia; simplified snowball sampling; n=1359 | Online survey | 21-30 [21-30 to 31-40] | 729 (68.5) | University graduated/post-graduated; junior/senior school graduated to university graduated/post-graduated | Monthly income (Indonesian Rupiah):  2.5-5 million [<2.5 million to 2.5-5 million]; student. | - |
| Lin et al. (2020)^57^ | 1^st^-19^th^ May 2020 | China; snowball sampling; n=3541 | Online survey; adult Chinese citizens who are members of the social network WeChat and contacts of the researchers. | 26-35 [26-35 to 36-45] | 1839 (51.9) | - | Average annually household income (CNY):  5001-120,000 [5001-120,000 to >120,000 | - |
| Mercadante et al. (2020)^58^ | 23^rd^- 29^th^ October 2020 | US; stratified sampling; n=525 | Online survey; Qualtrics Panel adult members | 30-49 [30-49 to 50-69] | 257 (49.0) | Some college; some high school to master’s degree or higher | Annual household income:  $21,000-$50,000 [<$20,000 to $51,000-$100,000] | 178 (33.9) |
| Mouchtouri et al. (2020)^59^ | 15^th^ April-2^nd^ May 2020 | Greece; randomised, geographically stratified sampling; n=1858 | Telephone survey conducted by interviewers over phone; Greek adults who owned a telephone. | 46-60 [18-45 to 61-92] | 1081 (58.8) | Bachelor’s degree; up to secondary school to Master or Doctor of philosophy degree | Middle [Low to Middle]; other occupation that is not civil servant or private sector employee. | - |
| Murphy et al. (2021)^60^ | UK: 23-28^th^ March 2020  Ireland: 31^st^ March-5^th^ April 2020 | UK and Ireland; quota Sampling; n=3066 (UK=2025, Ireland= 1041) | Online survey; adult participants of the COVID-19 Psychological Research Consortium Study | UK: 45-54 [25-34 to 55-64]  Ireland: 35-44 [25-34 to 55-64] | UK: 1047 (51.7)  Ireland: 536 (51.5) | UK: undergraduate; no qualifications to technical qualification  Ireland: undergraduate degree; no qualification to other technical qualification. | 2019 annual household income:  UK: £25,341–£38,740 [£15,491–£25,340 to £38,741–£57,930]; Full time (self)/employed.  Ireland: €30,000–€39,999 [€20,000–€29,999 to €40,000–€49,999]; Full time (self)/employed. | UK: 178 (8.8)  Ireland: 79 (7.6) |
| Prati et al. (2020)^61^ | April 2020 | Italy; Virtual snowball sampling; n=624 | Online survey; Italian adults active on the internet. | 32.31 [12.7] | 337 (54.0) | - | Perceived household economic resources in last 12 months:  Adequate [adequate to adequate]; employed. | 25 (4.0) |
| Reiter et al. (2020)^62^ | May 2020 | US; convenience sample; n=2006 | Online survey; survey panel (SSRS Glen Mills) | 50-64 [30-49 to 65 and older] | 1122 (56.0) | Some college; less than high school degree to college degree or more | Annual household income:  Less than $50,000 [Less than $50,000 to $50,000 to $89,999] | 659 (32.9) |
| Romer et al. (2020)^63^ | 17^th^-27^th^ March, 10^th^-21^st^ July 2020 | US; demographically stratified sampling; n= 840 | Online survey; NORC AmeriSpeak Panel | 45-59 [30-44 to 60+] | 435 (55.4) | Some college; high school or less to post-grad | Annual household income:  30-$85K [30-$85K to 85>200K] | 185 (22.0) |
| Sallam et al. (2021)^64^ | 14^th^ -18^th^ December 2020 | Jordan; convenience sampling; n=3414 | Online survey; social media and WhatsApp contacts of study authors | 22-26 [16-21 to 27-39] | 2299 (67.3) | Undergraduate; high school or less to postgraduate | Monthly income for residents in Jordan (data only for Jordan residents):  500–1000 JOD [Less than 500 JOD to More than 1000 JOD] | - |
| Sherman et al. (2020)^65^ | 14^th^- 17^th^ July 2020 | UK; quota sampling; n=1500 | Online survey; Profilic’s online research panel | 46.0 [15.8] | 765 (51.0) | Other or no qualifications; other or qualifications to degree or higher | Annual household income:  £30,000–£39,999 [£20,000–£29,999 to £50,000–£74,999]; Full-time and part-time. | 233 (15.5) |
| Ward et al. (2020)^66^ | April 2020 weekly | France; random stratified sampling; n=5018 | Online survey; IFOP research panel | 35-64 [<35 to 35-64] | 2629 (52.4) | Lower than high school degree; lower than high school degree to higher than Bachelor’s degree | - | - |
| Williams et al. (2020)^67^ | 1^st^-10^th^ April 2020 | UK; convenience sampling; n=527 | Online survey; 2 ongoing covid-19 studies for chronic respiratory disease & older adult vaccination | 59.5 [16] | 297 (56.7) | College; high school to postgraduate | Modal deprivation quintile (1 most deprived - 5 least deprived)= 3 [Range 2 to 4] | - |
| Wong et al. (2020)^68^ | 3^rd^-12^th^ April 2020 | Malaysia, snowball sampling; n=1159 | Online survey; social network platform contacts of authors | 31-40 [18-30 to 41-50] | 765 (66.0) | Tertiary; secondary or below to tertiary | Average month household income (MYR):  4001-8000 [2001-4000 to >8000]; Professional and managerial. | 42 (3.6) |
| Zeballos et al. (2021)^69^ | 29^th^ April- 9^th^ May 2020 | Bolivia; n=886 | Online survey; adult social media users | <25 [<25 to 25-45] | 577 (65.1) | Secondary schooling | Monthly income: NB. 1 minimum wage= 296USD  1-2 minimum wage [<1 minimum wage to >2 minimum wage]; secondary schooling. | - |

**Study Characteristics Table.** *Summary table detailing the characteristics of each included cross-sectional study.
LE= Egyptian Pound, CNY=Chinese Yuan Renminbi, UK= United Kingdom, US= United States, JOD= Jordanian Dinar, MYR= Malaysian Ringgit, USD= United States Dollar, K=thousand.*
