# Supplementary material for: Factors associated with COVID-19 vaccine intentions during the COVID-19 pandemic; a systematic review and meta-analysis of cross-sectional studies
Source: BMC Public Health. 2022 Sep 2;22:1667. doi: 10.1186/s12889-022-14029-4 (PMC9437387; doi:10.1186/s12889-022-14029-4)
Supplement: Supplementary file 4 — Additional file 4. Study Results Table. Summary table detailing the results of each included cross-sectional study. [file 12889_2022_14029_MOESM4_ESM.docx]

**Additional File 4: Study Results Table**

| **Author (Year of Publication)**  **Table.** *continued…* | **Vaccination Intention Question** | **Response Proportion % (n), [Proportion by sub-group where available %*]** | | | | | **Associated Factors** | **Additional Information** |
| --- | --- | --- | --- | --- | --- | --- | --- | --- |
|  |  | **Strongly Agree**** | **Agree**** | **Neither Agree nor Disagree**** | **Disagree**** | **Strongly Disagree**** |  |  |
| Abdelhafiz et al. (2020)^43^ | If there is an available vaccine for the virus, I am willing to get it | 73.0 (408) | 15.6 (87) | 4.30 (24) | 2.00 (11) | 8.60 (48) | - | - |
| Ali et al. (2020)^44^ | Suppose that a safe and effective coronavirus vaccine was available today. How likely are you to get yourself vaccinated? | 45.8 (2601),  [18-34yr= 49.5,  ≥35yr= 38.8] [F= 42.4, M=53.5] | 26.2 (1487),  [18-34yr= 25.7, ≥35yr =27.2] [F=27.2, M=23.9] | 18.1 (1026),  [18-34yr  =17.4, ≥35yr=19.3]  [F=19.2, M=15.5] | 6.60 (374),  [18-34yr =5.33, ≥35yr= 9.02]  [F=7.5, M=4.6] | 3.30 (189),  [18-34yr =2.14, ≥35yr =5.62]  [F=3.7, M=2.5] | Higher likelihood of receiving vaccine: younger subjects (<35), male, those working in healthcare). | Main sources of COVID-19 information via social media, WHO, TV and close family. |
| Alley et al. (2021)^45^ | If a new vaccine for COVID-19 was released that was proven to be safe and effective, I would get vaccinated immediately? | Survey 1= 64.2 (369)  Survey 2= 61.9 (356) | Survey 1= 23.0 (132)  Survey 2= 22.8 (131) | Survey 1= 8.00(46)  Survey 2= 10.1 (58) | Survey 1= 3.10 (18)  Survey 2= 3.80 (22) | Survey 1= 1.70 (10)  Survey 2= 1.40 (8) | More-likely hesitant than willing: female (OR 1.89, 95%CI 1.2-2.97, p<0.01)  Less likely to be willing: Survey 2 (OR 0.52, 95%CI 0.32-0.86, p<0.05). Technical studies diploma compared to Bachelor degree (OR 0.54, 95%CI 0.37-0.79, p<0.01). Consumed <1hr traditional media per day compared to those >3hr (OR 0.24, 95%CI 0.14-0.42) | - |
| Atwell et al. (2021)^46^ | If a COVID-19 vaccine were available today, would you get it? | 64.8 (848)  [F= 60.0, M= 71.0] | - | 27.1 (355)  [F= 31.0, M= 21.0] | - | 8.00 (105)  [F= 8.00, M= 8.00] | More likely to be hesitant than yes: Perceived COVID-19 less severe (RRR=1.54, 95%CI 1.25 to 1.91). Less trust in science (RRR=1.35, 95% CI 1.22 to 1.51). Less willing to have flu vaccine (RRR=2.14, 95% CI 1.81 to 2.55). Female (RRR= 1.97, 95% CI 1.47 to 2.64)  More likely to be hesitant than no: did not perceive COVID-19 to be a hoax (RRR=7.70, 95% CI 3.02 to 19.6)  Less likely to be hesitant than no: did not perceive covid-19 to be severe (RRR=0.74 ,95%CI 0.60 to 0.91). Did not have trust in science (RRR=0.88, 95% CI 0.79 to 0.65). Not willing to have flu vaccine (RRR=0.49, 95% CI 0.37 to 0.65) | Belief in disease severity and trust in science are most important factors that differ between those who are undecided and those accepting and resistant to covid-19 vaccine. |
| Bell et al.^47^ (2020) | If a new coronavirus (COVID-19) vaccine became available, would you accept the vaccine for yourself? | 55.8 (699) | 34.3 (429) | - | 7.40 (93) | 3.40 (43) | More likely to reject: lower household income (OR 2.08, 95%CI 1.31-3.30, p<0.001). BAME (OR 2.70, 95%CI 1.27-5.87, p=0.01). Homemaker (OR 3.03, 95%CI 1.78-5.12, p<0.001) | Accepting reasons: protecting self and others, if it’s recommended. Rejection reasons: vaccine safety concerns, new/rushed /not enough evidence. |
| Biasio et al. (2020)^48^ | Will you get vaccinated, if possible? | 92.0 (816) | - | - | - | 8.00 (69) | - | Willingness improved over 2 weeks from 88% to 96% corresponded with vaccine supplier announcement. Intention was significantly higher than influenza vaccine (p<0.01) |
| Detoc et al. (2020)^49^ | If a vaccine against the new coronavirus was available for next season, would you get vaccinated? | 23.8 (776) | 53.8 (1754) | 12.1 (394) | 6.40 (207) | 3.90 (128) | More likely to be willing: Male (OR 1.88, 95%CI 1.53-2.31, p<0.001). Health-care worker (OR 1.53, 95%CI 1.27-1.85, p<0.001). >30 years old (OR 1.53 for all age groups, p<0.001). Fear about covid-19 (OR 2.45, 95%CI 2.00-3.00, p<0.001). High perceived individual risk (OR 1.51, 95%CI 1.27-1.85, p<0.001) | Vaccine acceptance: Male 83.1% [95% CI 80.8–85.3%], Female 74.2% [95% CI 72.3–76.0%] (p < 0.005); general vaccine hesitant 61.9% (95% CI 59.1–64.7%); healthcare workers 81.5%, non-healthcare workers 73.7% (p < 0.005). |
| Fisher et al. (2020)^50^ | When a vaccine for the coronavirus becomes available, will you get vaccinated? | 57.6 (571)  [18-29yr= 49.6, 30-44yr=47.045-59yr= 52.0, ≥60yr= 76.5] [F= 51.6, M=64.0] | - | 31.6 (313)  [18-29yr= 40.5, 30-44yr=36.8, 45-59yr= 35.9, ≥60yr= 17.7]  [F= 36.4, M= 26.5] |  | 10.8 (107)  [18-29yr= 9.90, 30-44yr=16.2, 45-59yr= 12.1,≥60yr= 5.80] [F= 12.0, M= 9.50] | More likely to be hesitant or reject: No high school diploma (RRR 7.80, 95%CI 3.10-19.6). Black ethnicity compared to white (RRR 6.40, 95%CI 3.20-13.0). Not having flu vaccine last year (those received flu vaccine last year RRR 0.06, 95%CI 0.01-0.11) | Hesitancy reasons: concerns about vaccine safety and effectiveness, and a need for more information. Rejection reasons; antivaccine attitudes, beliefs, or emotions, lack of trust. |
| Garcia et al. (2020)^51^ | Today, would you be willing to pay for a vaccine that protects you against COVID-19? | 90.6 (513) | - | - | - | 9.40 (53) | - | - |
| Harapan et al. (2020)^52^ | Would you accept the covid-19 vaccine? | Yes WTP= 78.3 (1065)  Yes, only if free= 14.9 (203) | - | - | - | No even if free= 6.60 (91) | - | Monthly income, healthcare workers, those with higher perceived risk and Catholics have a higher WTP. |
| Lin et al. (2020)^53^ | If a COVID-19 vaccine were available on the market, would you take it? | 28.7 (1016) [F=29.9, M= 72.4] | 54.8 (1940) | - | 11.9 (421) | 4.50 (159) | More likely to be willing: Higher perceived overall health (OR 1.74, 95%CI 1.44-2.09)*.* Self-employed (OR 1.66, 95%CI 1.07-12.6). Service occupation (OR 1.61, 95%CI 1.05-2.49). Perception that vaccine decreases chances of getting COVID-19 (OR 3.14, 95% CI 2.05-4.83). Unconcerned about vaccine efficacy (OR 1.65, 95%CI 1.31-2.09) or vaccine side-effects (OR 1.52, 95%CI 1.25-1.84) | Rejection reasons; concerns of side-effects and efficacy. Accepting reasons:belief that vaccine decreases chances of getting COVID-19 and knowing vaccine has been taken by most of the public. |
| Mercadante et al. (2020)^54^ | If the COVID-19 vaccine becomes available, select one of the following statements that best fits your opinion:  a. I will get the COVID vaccine  b. I will not get the COVID vaccine | 66.7 (350) | - | - | - | - 1. (175) | More likely to say no if aged 18-49, household income of $20,000 or less, knowing someone affected by COVID-19 pandemic. | Higher general vaccine acceptance if elderly (70+), Bachelor’s degree, income between $101-150k. Lowest general vaccine acceptance amongst African Americans and income less than 20k. Rejection reasons; more concerned about the side-effects, belief that they do not need the vaccine. |
| Mouchtouri et al. (2020)^55^ | Should a vaccine be available for COVID-19, I will receive it | 28.4 (514) | 20.9 (379) | 31.8 (575) | 11.9 (216) | 7.00 (127) | - | Main sources of COVID-19 information are television and radio, internet. |
| Murphy et al. (2021)^56^ | If a new vaccine were to be developed that could prevent COVID-19, would you accept it for yourself? | UK= 69.0 (1397)  Ireland= 64.9 (676) | - | UK= 24.8 (503)  Ireland= 25.6 (266) | - | UK= 6.10 (125)  Ireland= 9.50 (99) | IRISH SAMPLE  More likely to be hesitant than willing: Female (OR 1.62, 95%CI 1.18-2.22). 35-44yrs (OR 2.00, 95%CI 1.06-3.75).  More likely to reject than accept: 35-44yrs (OR 3.33, 95%CI 1.17-9.47). Non-Irish ethnicity (OR 2.89, 95%CI 1.17-7.09).  More likely to be hesitant than reject: Non-Irish ethnicity (OR 2.76, 95%CI 1.05-7.19). Lower level of income (OR range 2.82-5.44, 95% CI ranged from 1.04, 7.66 to 1.98, 14.9)  UK SAMPLE  More likely to be hesitant than willing: Female (OR 1.43, 95%CI 1.14-1.8). <65 (p<0.05)  More likely to reject than accept: younger age (p<0.05). Lowest income (p<0.05). Pregnant (OR 2.36, 95%CI 1.03-5.40)  More likely to be hesitant than reject: Younger age | Psychological indicators of hesitancy/ resistance; less trust in science, professionals and state; negative attitudes to migrants; lower cognitive reflection; lower altruism, personality trait of agreeableness; higher levels of social dominance, authoritarianism, conspiratorial, religious beliefs and internal locus of control. More likely to be resistant vs hesitant if higher levels of conspiracy belief and less trust in science, professionals, and state. Resistors use significantly more social media for info and had less in serious news sources. |
| Prati et al. (2020)^57^ | Assume that your local health authority makes freely available a vaccine against SARS-CoV-2. Do you intend to get the vaccine?’ | 75.8 (473) | - | 10.1 (63) | - | 5.10 (32) | More likely to reject than willing: lower levels of worry (worry OR 0.83, 95%CI 0.70-0.99). Lower levels of institutional trust (trust OR 0.92, 95%CI 0.85-0.99)  More likely to be hesitant than willing: Older (OR 1.04, 95%CI 1.02-1.06). Belief in non-natural origin of virus (OR 2.96, 95%CI 1.39-6.30) | - |
| Reiter et al. (2020)^58^ | How willing would you be to get the COVID-19 vaccine if it was free or covered by health insurance? | 48.0 (963)  [18-29yr= 71.0, 30-49yr=65.050-64yr =64.0, 65+yr= 76.0] [F= 64.0, M= 75.0] | 21.0 (421) | 17.0 (341) | 5.00 (100) | 9.00 (181)  [18-29yr= 29.0, 30-49yr= 35.0, 50-64yr= 36.0, 65+yr=24.0] [F=36.0, M= 25.0] | More Willing: Higher income (RR 1.09, 95%CI 1.02-1.16). Private health insurance (RR 1.12, 95%CI 1.00-1.26). Personal history of COVID-19 infection (RR 1.12, 95%CI 1.01-1.27). Thought their healthcare provider would recommend vaccination (RR 1.73, 95%CI 1.49-2.02). Higher perceived likelihood of getting COVID-19 (RR 1.05, 95%CI 1.01-1.09). Higher perceived severity of covid-19 infection (RR=1.08, 95%CI 1.04-1.11). Higher perceived effectiveness of COVID-19 vaccine (RR 1.46, 95%CI 1.4-1.52)  Less willing  Female (RR 0.91, 95%CI 0.87-0.96). Non-Latinx Black (RR 0.81, 95%CI 0.74-0.90). Higher level of perceived harms of covid-19 vaccine (RR 0.95, 95%CI 0.92-0.98) | Factors in vaccine decision; efficacy, if doctor recommends vaccine, health history, number. people infected with COVID-19, age, future travel (those more vaccine willing were less likely to indicate potential side-effects mattering in their decision). Maximum WTP $0(30%), $1-$19 (15%), $20-$49 (20%), $50-$99 (14%), $100-$199 (10%), and $200+ (11% |
| Romer et al. (2020)^59^ | If there were a vaccine that protected you from getting the coronavirus, how likely, if at all, would you be to decide to be vaccinated? | 59.6 (501) | - | 25.9 (217) | - | 14.5 (122) | Belief in conspiracy beliefs reduced vaccination intention (assessed by belief in MMR harm). | Positive relation between social media use and perceptions of MMR harm. Education and white ethnicity reduced belief in MMR harm. Vaccine intentions and conspiracy belief positively correlated over time. |
| Sallam et al. (2021)^60^ | Will you get the coronavirus vaccine when available? | 40.3 (980)  [F=23.9, M=38.6] | - | - | - | 59.7 (1454)  [F=76.1, M=61.4] | More willing: Male (p<0.001). Higher educational level (p<0.001). History of chronic disease (p=0.009). | Most likely to believe vaccine conspiracies if; female, lower education, <1000 JOD. Least likely if news from doctors, scientists and scientific journals.  Belief prevalence: COVID-19 is a man-made virus (59.5%), COVID-19 is man-made to force everyone to get the vaccine (40.2%), COVID-19 is a way to implant microchips into people to control them (27.7%), COVID-19 vaccines will cause infertility (23.4%) |
| Sherman et al. (2020)^61^ | When a coronavirus vaccination becomes available to you, how likely is it you will have one? | 64.0 (960) | - | 26.9 (403) | - | 9.10 (137) | More willing (p<0.01): Older. Having influenza vaccine last year. Greater perceived risk of COVID-19 to people in UK. Less beliefs in side-effects or unsafe vaccine. Positive COVID-19 beliefs. Less belief that only those at risk of serious illness should be vaccinated. Greater perceived information sufficiency to make informed decision. | - |
| Ward et al. (2020)^62^ | Would you agree to get vaccinated if a vaccine against the COVID-19 was available? | 76.0 (3814)  [<35yr= 66.9, 35-64yr=74.2>64yr=89.3] [F=72.9, M=79.4] | - | - | - | 24.0 (1204)  [<35yr= 33.1, 35-64yr=25.8, >64yr=10.7] [F=27.1, M=20.6] | More likely to reject than accept: Female (male OR 0.75, 95%CI 0.65-0.86, p<0.001)*.* <35 years old (OR 1.24, 95%CI 1.06-1.45, p<0.001). Lower COVID-19 related concern (higher OR 0.54, 95%CI 0.45-0.66, p<0.001) | Rejection reasons; against general vaccines (27.6% of refusers- especially women), vaccine produced in a rush is dangerous (64.4%), vaccine useless as covid-19 is harmless (9.6%- especially men). |
| Williams et al. (2020)^63^ | If a vaccine for coronavirus becomes available, would you want to receive it? | 58.4 (307) | 27.2 (143) | 7.20 (38) | 1.70 (9) | 5.50 (29) | Positive correlation with belief that COVID-19 outbreak will continue for a long time and vaccination willingness (p<0.05). Negative correlation with belief that media has over-exaggerated risk of catching COVID-19 (p<0.05) | Rejection reasons; concerns over vaccine safety. Accepting reasons; higher perceived severity of disease and health consequences to others. |
| Wong et al. (2020)^64^ | If a vaccine against COVID-19 infection is available in the market, would you take it? | 48.2 (559)  [18-30yr= 95.9, 31-40yr=94.4, 41-50yr= 93.1, >50yr= 92.5] [F= 94.4, M= 94.2] | 29.8 (345) | 16.3 (189) | 3.3 (38) | 2.4 (28)  [18-30yr= 4.1, 31-40yr= 5.6, 41-50yr= 6.9,>50yr= 7.5] [F=5.6, M= 5.8] | More Willing: Males (OR 1.44, 95%CI 1.11-1.87, p<0.01). Belief that vaccine decreases chance of COVID-19 infection (OR 2.51, 95%CI 1.19-5.26, p<0.05). Belief that vaccine makes them less worried about COVID-19 (OR 2.19, 95%CI 1.03-4.65, p<0.05). No worried about vaccine side-effects (OR 1.81, 95%CI 1.34-2.44, p<0.001). Disagreement that only take vaccine if taken by majority of public (OR 1.49, 95%CI 1.12-1.98, p<0.01). Belief in possibility of getting COVID-19 (OR 1.36, 95%CI 1.04-1.79, p<0.05) | Mean max. WTP MYR$134.0 (SD±79.2). Higher WTP if; tertiary education, professional/managerial, higher income, not concerned about vaccine affordability or if kosher. >50% concerned about whether the COVID-19 vaccine is halal. |
| Zeballos et al. (2020)^65^ | Would you use a COVID-19 vaccine if it were available? | 54.3 (481) | - | 29.8 (264) | - | 15.9 (141) | More Willing: Higher risk perception of COVID-19 (p<0.001). Higher risk perception if female (p<0.001) and higher exposure to COVID-19 info on social media (p=0.002) | - |

**Study Results Table.** *Summary table detailing the results of each included cross-sectional study.
*Sub-groups include sex, age, and location.
**’Strongly Agree’ includes Yes/Very Likely/Accepting/Strongly Agree. ‘Agree’ includes Somewhat Likely / Agree/ Unsure but leaning towards Yes / Probably Yes. ‘Neither agree nor disagree’ includes Not Sure / Neither Agree nor Disagree / Neutral / I don’t know/ Maybe / Hesitant. ‘Disagree’ includes Somewhat Unlikely/ Disagree / Unsure but Leaning towards No/ Probably No. ‘Strongly Disagree’ includes No/ Very Unlikely/ Strongly Disagree / Resistant.
Yr= year, M=Male, F=Female, WHO= World Health Organisation, TV= Television, OR=Odds Ration, CI=Confidence Interval, RRR= Relative Risk Ratio, BAME= Black, Asian and Minority Ethnic, k=thousand, RR= Relative Risk, WTP= Willingness to Pay, JOD=Jordanian Dinar, MMR= Measles, Mumps and Rubella, MYR= Malaysian Ringgit.*
