# Supplementary material for: Factors associated with COVID-19 vaccine intentions during the COVID-19 pandemic; a systematic review and meta-analysis of cross-sectional studies
Source: BMC Public Health. 2022 Sep 2;22:1667. doi: 10.1186/s12889-022-14029-4 (PMC9437387; doi:10.1186/s12889-022-14029-4)
Supplement: Supplementary file 5 — Additional file 5. AXIS Summary Table [41]. Summary table of results of the appraisal of cross-sectional studies (AXIS) for each study. [file 12889_2022_14029_MOESM5_ESM.docx]

|  | Abdelhafiz (2020)^43^ | Ali (2020)^44^ | Alley (2021)^45^ | Atwell (2021)^46^ | Bell (2020)^47^ | Biasio (2020)^48^ | Detoc (2020)^49^ | Fisher (2020)^50^ | Garcia (2020)^51^ | Harapan (2020)^52^ | Lin (2020)^53^ | Mercadante (2020)^54^ | Mouchtouri (2020)^55^ | Murphy(2021)^56^ | Prati (2020)^57^ | Reiter (2020)^58^ | Romer (2020)^59^ | Sallam (2021)^60^ | Sherman (2020)^61^ | Ward (2020)^62^ | Williams(2020)^63^ | Wong (2020)^64^ | Zeballos(2021)^65^ |
| --- | --- | --- | --- | --- | --- | --- | --- | --- | --- | --- | --- | --- | --- | --- | --- | --- | --- | --- | --- | --- | --- | --- | --- |
| Clear Aims and Objectives? | ✓ | ✓ | ✓ | ✓ | ✓ | ✓ | ✓ | ✓ | ✓ | ✓ | ✓ | ✓ | ✓ | ✓ | ✓ | ✓ | ✓ | ✓ | ✓ | ✓ | ✓ | ✓ | ✓ |
| Appropriate Study Design for the Aims? | ✓ | ✓ | ✓ | ✓ | ✓ | ✓ | ✓ | ✓ | ✓ | ✓ | ✓ | ✓ | ✓ | ✓ | ✓ | ✓ | ✓ | ✓ | ✓ | ✓ | ✓ | ✓ | ✓ |
| Justified Sample Size? | ✓ | X | X | X | X | X | X | X | X | X | ✓ | ✓ | ✓ | ✓ | ✓ | X | ✓ | X | ✓ | X | X | X | X |
| Target population clearly defined? | ✓ | X | ✓ | ✓ | ✓ | ✓ | ✓ | ✓ | ✓ | ✓ | X | ✓ | ✓ | ✓ | X | ✓ | ✓ | ✓ | ✓ | ✓ | ✓ | ✓ | ✓ |
| Appropriate sample frame for representative sample? | X | X | ✓ | ✓ | X | X | X | ✓ | ✓ | ? | X | ✓ | ✓ | ✓ | X | ✓ | ✓ | X | ✓ | ✓ | X | X | X |
| Selection Process to select representative subjects? | X | X | X | X | ✓ | X | X | ✓ | ✓ | X | X | ✓ | ✓ | ✓ | ✓ | X | ✓ | X | ✓ | ✓ | X | X | ? |
| Addressed and categorised non-responders? | X | ✓ | X | ✓ | X | X | X | ✓ | X | X | ✓ | X | ✓ | X | ✓ | X | ✓ | X | X | X | X | X | ? |
| Appropriate outcomes for the aims? | ✓ | ✓ | ✓ | ✓ | ✓ | ✓ | ✓ | ✓ | ✓ | ✓ | X | ✓ | ✓ | ✓ | ✓ | ✓ | ✓ | ✓ | ✓ | ✓ | ✓ | ✓ | ✓ |
| Trialled/ piloted/ published survey instruments? | ✓ | ✓ | X | X | ✓ | ✓ | X | ✓ | ✓ | ✓ | ✓ | ✓ | ✓ | ✓ | ✓ | ✓ | X | ✓ | ✓ | X | X | ✓ | ✓ |
| Pre-determined statistical significance level? | ✓ | X | X | ✓ | ✓ | ✓ | ✓ | ✓ | X | ✓ | ✓ | ✓ | ✓ | ✓ | ✓ | ✓ | ✓ | ✓ | ✓ | ✓ | X | ✓ | ✓ |
| Repeatable methods? | ✓ | X | ✓ | ✓ | ✓ | ✓ | X | ✓ | ✓ | X | ✓ | ✓ | ✓ | ✓ | ✓ | ✓ | X | ✓ | ✓ | ✓ | ✓ | ✓ | X |
| Basic data described? | ✓ | ✓ | ✓ | X | ✓ | X | ✓ | ✓ | ✓ | ✓ | ✓ | ✓ | ✓ | ✓ | ✓ | ✓ | ✓ | ✓ | ✓ | ✓ | ✓ | ✓ | ✓ |
| Response rate raise concerns over Non-response bias? | ? | ✓ | ✓ | ✓ | X | ? | ✓ | X | X | ? | ✓ | X | ✓ | ? | X | X | ✓ | ✓ | ? | ? | ✓ | ✓ | ✓ |
| Non-responders described? | ? | ✓ | ✓ | ✓ | X | X | X | X | X | X | ✓ | X | ✓ | X | ✓ | X | ✓ | X | X | X | ✓ | X | X |
| Results internally consistent? | ✓ | ✓ | X | ✓ | ✓ | X | ✓ | X | X | X | ✓ | ✓ | ✓ | X | ✓ | ✓ | X | ✓ | ✓ | ✓ | X | ✓ | ✓ |
| All analyses presented were described in methods? | ✓ | X | ✓ | ✓ | X | X | ✓ | ✓ | ✓ | ✓ | ✓ | ? | ✓ | ✓ | ✓ | ✓ | ? | ✓ | ✓ | ✓ | ✓ | ✓ | ✓ |
| Discussions and conclusions justified by results? | X | ✓ | ✓ | X | ✓ | ✓ | ✓ | ✓ | X | ✓ | ✓ | ✓ | ✓ | ✓ | ✓ | ✓ | ✓ | ✓ | ✓ | X | ✓ | ✓ | X |
| Limitations discussed? | ✓ | ✓ | ✓ | X | ✓ | ✓ | ✓ | X | X | ✓ | ✓ | ✓ | ✓ | ✓ | ✓ | ✓ | ✓ | ✓ | ✓ | X | ✓ | ✓ | ✓ |
| Funding sources or conflicts of interest? | X | X | X | X | ✓ | X | X | X | X | X | X | X | X | X | X | X | X | X | X | X | X | X | X |
| Ethical approval or consent attained? | ✓ | ✓ | ✓ | ✓ | ✓ | ✓ | ✓ | ✓ | ? | ✓ | ✓ | ✓ | ✓ | ✓ | ✓ | ✓ | ✓ | ✓ | ✓ | ✓ | ✓ | ✓ | ✓ |

**Additional File 5: AXIS Summary Table^41^.**

**AXIS Summary Table.** *Summary table of results of the appraisal of cross-sectional studies (AXIS) for each study.*✓*=Yes,X=No,?= Unsu*
