# Supplementary material for: Factors associated with COVID-19 vaccine intentions during the COVID-19 pandemic; a systematic review and meta-analysis of cross-sectional studies
Source: BMC Public Health. 2022 Sep 2;22:1667. doi: 10.1186/s12889-022-14029-4 (PMC9437387; doi:10.1186/s12889-022-14029-4)
Supplement: Supplementary file 6 — Additional file 6. Data Extraction Form. A copy of the data extraction form used to extract data from each study included in the review. [file 12889_2022_14029_MOESM6_ESM.docx]

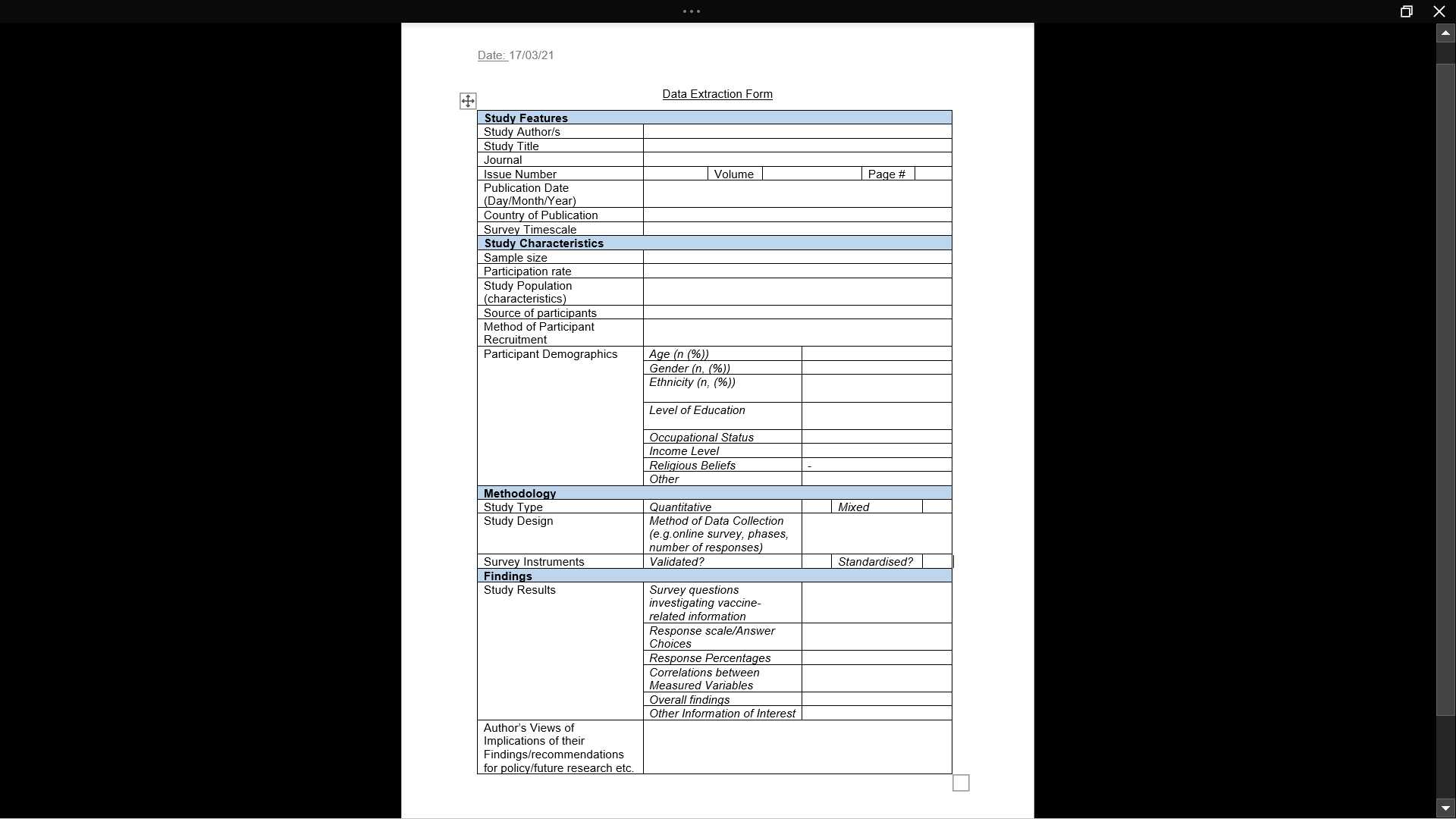
**Additional File 6: Data Extraction Form.** A copy of the data extraction form used to extract data from each study included in the review.
