# Supplementary material for: Factors associated with COVID-19 vaccine intentions during the COVID-19 pandemic; a systematic review and meta-analysis of cross-sectional studies
Source: BMC Public Health. 2022 Sep 2;22:1667. doi: 10.1186/s12889-022-14029-4 (PMC9437387; doi:10.1186/s12889-022-14029-4)
Supplement: Supplementary file 7 — Additional file 7. AXIS Form. A copy of the AXIS form used for the assessment of bias and quality of each study included in the review. [file 12889_2022_14029_MOESM7_ESM.docx]

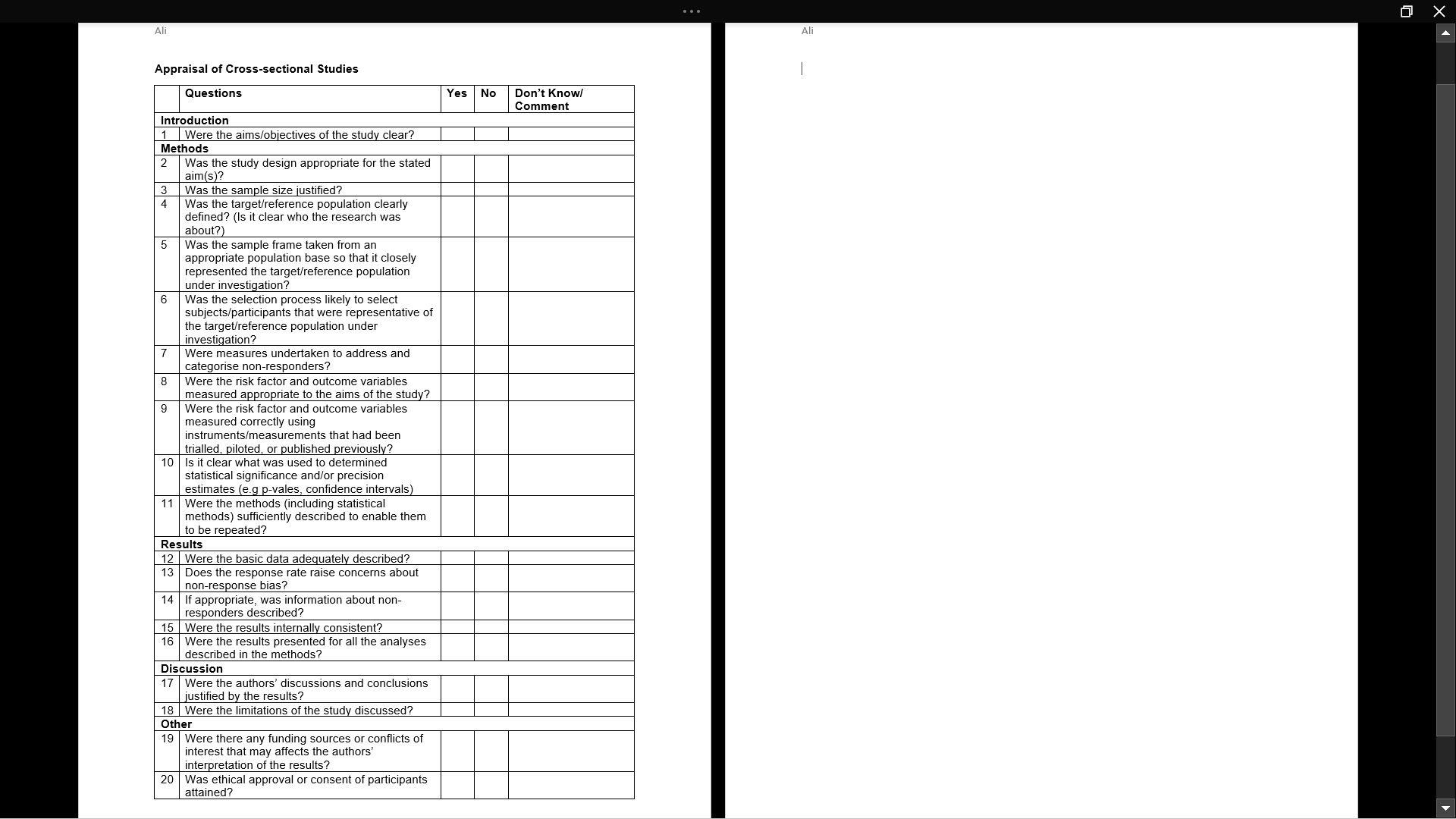
**Additional File 7: AXIS Form.** A copy of the AXIS form used for the assessment of bias and quality of each study included in the review.
